# Tryptophan As a New Member of RNA‐Induced Silencing Complexes Prevents Colon Cancer Liver Metastasis

**DOI:** 10.1002/advs.202307937

**Published:** 2024-06-20

**Authors:** Fangyi Xu, Yi Ren, Yun Teng, Jingyao Mu, Jie Tang, Kumaran Sundaram, Lifeng Zhang, Juw Won Park, Jae Yeon Hwang, Jun Yan, Gerald Dryden, Huang‐Ge Zhang

**Affiliations:** ^1^ Brown Cancer Center University of Louisville Louisville KY 40202 USA; ^2^ Department of Central Laboratory Cancer Center The affiliated Huaian No. 1 People's Hospital of Nanjing Medical University Huai'an 223300 China; ^3^ Department of Breast and Thyroid Surgery The affiliated Huaian first People's Hospital of Nanjing Medical University Huaian Jiangsu 223300 China; ^4^ Department of Computer Science and Engineering University of Louisville Louisville KY 40202 USA; ^5^ Robley Rex Veterans Affairs Medical Center Louisville KY 40206 USA; ^6^ Department of Microbiology & Immunology University of Louisville Louisville KY 40202 USA

**Keywords:** Ago2, amino acids, caprin1, colon cancer, liver metastasis, microRNAs, miR‐193a, RISC, tryptophan

## Abstract

Essential amino acids (EAA) and microRNAs (miRs) control biological activity of a cell. Whether EAA regulates the activity of miR has never been demonstrated. Here, as proof‐of‐concept, a tryptophan (Trp, an EAA) complex containing Argonaute 2 (Ago2) and miRs including miR‐193a (Trp/Ago2/miR‐193a) is identified. Trp binds miR‐193a‐3p and interacts with Ago2. Trp/Ago2/miR‐193a increases miR‐193a‐3p activity via enhancing Argonaute 2 (Ago2) RNase activity. Other miRs including miR‐103 and miR‐107 in the Trp complex enhance miR‐193a activity by targeting the same genes. Mechanistically, the Trp/Ago2/miR‐193a complex interacts with Trp‐binding pockets of the PIWI domain of Ago2 to enhance Ago2 mediated miR activity. This newly formed Ago2/Trp/miR‐193a‐3p complex is more efficient than miR‐193a‐3p alone in inhibiting the expression of targeted genes and inhibiting colon cancer liver metastasis. The findings show that Trp regulates miR activity through communication with the RNA‐induced silencing complexes (RISC), which provides the basis for tryptophan based miR therapy.

## Graphical abstract

Proposed model of amino acid modulates the miR activity by directly binding to miR, subsequently enhancing Ago2 RNase activity via binding to Trp bound pockets of Ago2 for increasing miR mediated inhibition of expression of targeted genes, leading to inhibition of colon cancer liver metastasis. The studies reveal that tryptophan/miR/Ago2 complex inhibits mouse colon cancer liver metastasis.

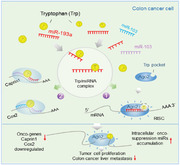

## 1 Introduction

Tryptophan (Trp) is one of the amino acids in building proteins that carry out life's essential actions. Beyond its role in protein biosynthesis, recent studies indicated that tryptophan is involved in the regulation of immunity, neuronal function and intestinal homeostasis.^[^ ^1^ ^]^ Imbalances in tryptophan and tryptophan mediated metabolism contribute to development of diseases ranging from cancer to neurodegenerative disease.^[^ ^2^, ^3^ ^]^ These studies have shed light on the important role of systemic tryptophan homeostasis. Higher plasma tryptophan levels are associated with lower colon cancer risk, while increased metabolism of tryptophan (Trp), serotonin and kynurenine, are associated with a higher risk of colon cancer.^[^ ^4^ ^]^ Whether higher systemic concentrations of Trp could play a role in the inhibition of disease development, such as colon cancer metastasis, is unclear. Addressing this question could shed light on the molecular mechanisms used by Trp and Trp mediated cellular pathways that contribute to prevention/treatment of disease.

The expression levels of mRNAs are sensitive to changes in nutrient status and diet‐derived tryptophan regulates the expression of specific mRNAs in human tissues.^[^ ^1^, ^5^ ^]^ It is well known that the levels of mRNA expression are regulated by miRs. Whether tryptophan mediated modulation of expression of mRNA is through regulation of miRNA (miR) activity is not known. Mature miR, which enters the RNA‐induced silencing complexes (RISC), is capable of binding to partially complementary sequences within the 3′UTR of the target mRNA. Ago2 controls miR activity by providing the RNase cleavage activity that targets mRNAs that complement the guiding miR. Ago2 is the only protein component required for RISC activity.^[^ ^6^ ^]^ Ago2 PIWI domain possesses a Trp binding region, composed of three Trp‐binding pockets. This domain allows Ago2 to interact with diverse unstructured Trp (GW)‐rich proteins in RISC.^[^ ^7^ ^]^ Interactions between Ago2 and GW‐rich TNRC6B in RISC promote the assembly of massive complexes and control of Ago2 RNase activity.^[^ ^7b,c^ ^]^ Not only does Trp residue in a protein enhance miR activity,^[^ ^7b,c^ ^]^ but Trp can bind to RNA via both its α‐carbon groups and side chain.^[^ ^8^ ^]^ This is consistent with frequent RNA sites that bind several aromatic amino acids, but not other types of side chains.^[^ ^9^ ^]^ Whether Trp can bind miR and further enhance miR activity via regulation of miR associated Ago2 RNase activity has not been studied. The finding that tryptophan can regulate miR activity is significant since miRs are capable of modulating at the translational level up to 60% of the protein‐coding genes in the human genome.^[^ ^10^ ^]^ miRs are found in different organisms and are involved in diverse biological settings. In humans, they play a crucial role in controlling cell development, function, and metabolism by regulating gene expression. miRs are associated with cancer, cardiovascular, and inflammatory diseases, as well as a broad range of neurodevelopmental and autoimmune diseases. Therefore, our findings open a new avenue to use Trp as a potential therapeutic agent to enhance miR therapy in different types of disease.

In this study, we investigated a possible role of Trp beyond building proteins and Trp mediated metabolism pathways. First, we discovered the Trp/miR complex. miR array analysis of Trp pulldown complex revealed an unexpected role of Trp as an organizer for assembling the Trp/Ago2/miRs complex. Further, we took advantage of the single‐strand gel shift (SSGS) technology developed recently^[^ ^11^ ^]^ as a means to identify the miR‐193a‐3p that can bind to Trp in the Trp/Ago2/miRs complex. We demonstrate that Trp can directly bind to miR‐193a‐3p that can form complexes with other miRs. The formation of Trp/Ago2/miRs complex generates new biological effects.

We then determined whether miR‐193a‐3p in the context of the Trp/miRs complex has a stronger activity than miR‐193a‐3p alone in terms of inhibition of expression of its targeted gene. We chose caprin1^[^ ^12^ ^]^ which is targeted by miR‐193a‐3p. We discovered that the formation of the Trp/miR‐193a‐3p complex is required to inhibit metastasis of colon cancer to the liver via targeting the oncogene caprin1 gene. Specifically, interaction of Ago2 with Trp/miR‐193a‐3p enhances Ago2 mediated miR‐193a‐3p activity, inhibiting the expression of caprin1. Collectively, our discovery demonstrates how the amino acid Trp can prevent cancer metastasis by miR communication via defined molecular mechanisms. Specially, we show that Trp regulates miR activity through enhancing the Ago2 RNase activity.

## 2 Results

### 2.1 A Lower Level of Trp Alters the miR Profile in the Trp/miR Complex

**Figure 1:**
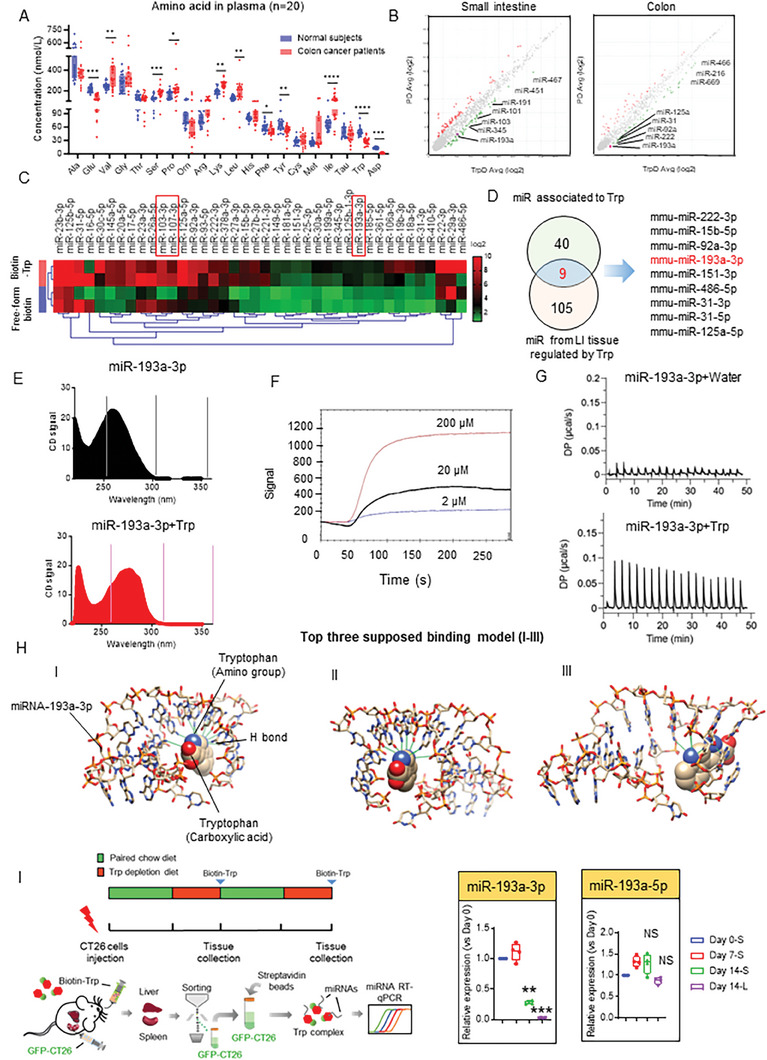
A lower level of Trp alters the miR profile in the Trp/miR complex. A). Dots represent concentration of a specific amino acid in plasma of colorectal cancer patients and healthy subjects (n = 20). The concentrations of amino acid (Alanine, Glutamine, Valine, Glycine, Threonine, Serine, Proline, Ornithine, Arginine, Lysine, Leucine, Histidine, Phenylalanine, Tyrosine, Cysteine, Methionine, Isoleucine, Taurine, Tryptophan, Asparagine) in the healthy subjects or in colon cancer patients are shown, P values were calculated using an ANOVA test. ^*^ *p* <0.05, ^**^ *p* < 0.01, ^***^ *p* < 0.001. ^****^ *p* <0.0001. B). C57BL/6 mice (n = 5 per group) were treated with Trp‐free diet (TrpD) or a regular paired diet (PD, tryptophan, 0.3%, w/w) for 3 days. Volcano plots from differential miR expression analysis in small intestine tissue (SI) and colon tissue (LI) from mice fed the Trp‐free diet or paired diet are shown. The grey dots represent the level of miRs in mice fed the TrpD or the PD are similar (*p*>0.05). Red dots represent the level of miRs in mice fed with TrpD are significantly lower than it in PD diet (*p*<0.05). C). CT26 colon tumor cells were cultured under Trp‐free cell culture medium conditions for 12 h, then treated with biotin labeled Trp (Biotin‐Trp, 200 µM) or free‐form biotin in Trp deficient media for an additional 12 h. Trp complexes were pulled down with streptavidin beads and the miR interacting with Trp was isolated. miR levels from CT26 cells treated with/without biotin labeled Trp (n = 2) were analyzed using a miR chip and the data generated from the miR chip are presented as a heatmap plot. The miRs with red color rectangles indicate comparable levels of miRs data generated from both samples. D). Venn diagram illustrates that the 9 overlapping miRs exhibit altered expression in mice fed the TrpD diet (B) and CT26 cells cultured in Trp‐deficient media (C). E). CD spectra of miR‐193a‐3p (100 nM) in the absence (black) or presence (red) of Trp (200 µM). More details can be found in the Methods section. F). The surface plasmon resonance (SPR) assay was performed to determine Trp binding to miR‐193a‐3p (10 µM). The signal output is directly related to changes in binding capacity on the sensor surface. The three curves represent Trp signals with increasing concentration: 2 µM (light blue), 20 µM (black), and 200 µM (red). G). Representative ITC experiments for Trp binding to miR‐193a‐3p. Apparent Kd, entropy (ΔS), and enthalpy (ΔH) values for individual experiments are determined; Trp (200 µM) binding to the miR‐193a‐3p in HEPES buffer (pH 7.2). Details can be found on Method section. H). 3D structure modeling prediction of Trp and miR‐193a‐3p was simulated in silico. The miR‐193a‐3p sequence was submitted to the online HDOCK SERVER and analyzed by Chimera 1.16 software. Structure pattern diagram representing the top three fitting binding models of Trp to miR‐193a‐3p. I). Schematic plot showed the timeline of colon metastatic liver in a mouse model. BALB/c mice (n = 6) were injected intrasplenically with GFP‐CT26 cells (1 × 10^6^ per mouse). At day 4 or 11 after the GFP‐CT‐26 injection, the mice were provided the Trp‐free diet for 3 days and gavage given biotin labeled Trp (300 mg k^−1^g) before being sacrificed. GFP‐CT26 cells from spleen or liver were isolated by FACS sorting. Total miR was extracted and pulled down with streptavidin beads (20 µg RNA in 100 µL beads) to isolate the Trp complex. The quantitative reverse transcription polymerase chain reaction (RT‐qPCR) was performed to quantify the level of miR‐193‐3p and miR‐193a‐5p. The value of spleen on Day 7 and 14 (Day 0, 7, 14‐S) and of liver on Day 14 (Day 14‐L) was normalized to the miR level at Day 0. *p*‐values were calculated using an ANOVA test. ^**^ *p* < 0.01, ^***^ *p* < 0.001.

Amino acids (AA) provide building blocks for cancer cell growth. Cellular adaptation to AA availability is a prime strategy for optimizing tumor cell growth. We analyzed plasma AA levels in colon cancer patients (**Figure** **1**A). Interestingly, the levels of tryptophan (Trp), Asp, Tyr, Glu, and Phe were significantly lower than those from healthy subjects (Figure 1A) while the levels of Val, Ser, Lys, Leu, Met, and Ile are higher and the levels of other AAs including Ala, Gly, Thr, Pro, Orn, Arg, His, Cys, and Tau were no different (Figure 1A, (Tables S1–S5, Supporting Information). Further statical analysis indicate that among these five amino acids decreased in colon cancer patients, Trp has the most reduction and lowest value among these five AAs. The conclusion that circulating Trp in colon cancer patients is lower is also supported by the fact that HPLC analysis showed that concentrations of Trp were lower in tumor tissue compared to adjacent normal tissue of patients in all stages of colon cancer (Tables S1–S5, Supporting Information). Therefore, as a proof of concept, we selected Trp for further investigating its role in this study. RNA can bind amino acids,^[^ ^8^, ^9^, ^13^ ^]^ and Trp interacts with RNA.^[^ ^9b^ ^]^ To further determine whether RNA associated with Trp can modulate tumor cell growth, CT26 cells were treated with biotin labeled Trp complexes. Using streptavidin coated beads, we isolated biotin labeled Trp complexes from large intestine (LI) tissue of BALB/c mice gavage‐given biotin labeled Trp (300 mg k^−1^g). We then packed the isolated complexes in dioleoylphosphatidylethanolamine (DOPE) liposomes. The effects of the Trp complex on CT26 tumor cell growth were evaluated. The reason for choosing CT26 cells was because CT26 cells are well‐represented in the literature and accurately mimick the human tumor microenvironment.^[^ ^14^ ^]^ The growth of CT26 cell treated with Trp complexes (Figure S1A, Supporting Information) was significantly inhibited whereas the inhibitory effect on CT26 cell growth was abolished when the complex was pre‐treated with RNase but not DNase (Figure S1A, Supporting Information), which suggest that Trp associated RNA plays a crucial role in inhibiting tumor cell growth.

An estimated 2588 mature miRs regulate over 60% of the human genes and participate in every aspect of cellular activity in cell growth and death. While the interactions of Trp with RNA are documented,^[^ ^8^, ^9^ ^]^ whether the level of miR expression is regulated by Trp is not known. According to our miR chip array data (Figure 1B), the miR profile generated from small intestine (SI) and large intestine (LI) of C57BL/6 mice fed a Trp‐free diet (TrpD, tryptophan 0%, w/w) was different from mice fed a regular paired diet (PD, tryptophan 0.3%, w/w) (Figure 1B, GSE212633 and GSE212607), suggesting that Trp alters the expression of miR. To further validate whether the level of tryptophan influences the expression of miRs, CT26 cells were treated with tryptophan depleted media compared to tryptophan sufficient media for 48 h. Then, the expression of miR‐193a which was selected based on the data presented in Figure 1B was quantified using the quantitative reverse transcription polymerase chain reaction, (RT‐qPCR). We found that the higher concentration of Trp in the CT26 media, the higher level of expression of mature miR‐193a‐3p in the Trp treated CT26 cells (Figure S1B, Supporting Information, left panel), but not pre‐miR‐193a (Figure S1B, Supporting Information, middle panel) and miR‐193a‐5p (Figure S1B, Supporting Information, right panel). The increased level of miR‐193a‐3p is unlikely due to Trp affecting miR‐193a‐3p synthesis since the RT‐qPCR results indicated that Trp treatment has no effect on the expression of premature miR‐193a (Figure S1B, Supporting Information, middle panel).

Indoleamine 2,3‐dioxygenase 1 (IDO1) is one of the tryptophan enzymes that catalyzes the conversion of tryptophan into kynurenine.^[^ ^15^ ^]^ Therefore, we tested whether the level of tryptophan could be modulated by IDO1. The results indicated that higher levels of Trp are detected in the large intestine (LI) of IDO1 KO mice compared to WT mice (Figure S1C,D, Supporting Information). Interestingly, IDO1 KO mice also had higher levels of miR‐193a‐3p in LI tissue than WT mice (Figure S1E, Supporting Information). In summary, our results indicate that the levels of Trp and miR‐193a‐3p are modulated by IDO1 expressed in LI tissue.

To test whether Trp is associated with the miR complex, CT26 colon tumor cells were cultured under Trp‐free cell culture medium conditions for 12 h, then treated with biotin labeled Trp (Biotin‐Trp) or free form biotin in Trp deficient media for an additional 12 h. We isolated and pulled down total miRs using streptavidin beads. We analyzed the miR profile associated with Trp using a miR microchip array. Heatmap analysis data indicates that certain miRNAs are pulled down with biotin labeled Trp but not with the free‐form of biotin (Figure 1C; Table S6, Supporting Information). We then confirmed these results using RT‐qPCR with five randomly chosen miRs (miR‐193a‐3p, miR‐378a‐3p, miR‐29a‐3p, miR‐106a‐5p and miR‐17‐5p) (Figure S1F, Supporting Information).

Collectively, the results suggest that Trp interacts with miR and the level of these miRs is altered as a result of depletion of Trp. We then determined which miR listed in Figure 1C can directly bind to Trp using the single‐strand gel shift (SGSS) assay we have described.^[^ ^11^ ^]^ Nine miRs were selected for the SGSS test based on their altered expression in the intestine that was influenced by tryptophan. These miRs are associated with the tryptophan complex (Figure 1D), and shift was observed in the group of Trp mixed with miR‐193a‐3p. A shift was not detected in the other miRs, which suggest that only miR‐193a‐3p can bind directly to Trp (Figure S1G,H, Supporting Information). Thus, as proof of concept, miR‐193a‐3p was selected as a representative miR for further testing our hypothesis that tryptophan is a new member of RNA‐induced silencing complexes that can prevent colon cancer liver metastasis. In addition, the reason for choosing the mouse colon cancer model was because miR‐193a‐3p inhibits CT26 colon tumor cell growth and metastasis via inhibiting the expression of the oncogene caprin1.^[^ ^11^ ^]^

To further demonstrate that Trp directly and specifically binds to miR‐193a‐3p, three different independent approaches were performed as described below. Circular dichroism (CD) spectra of nucleic acids are commonly used to provide a signature for a given secondary structural change.^[^ ^16^ ^]^ We observed that there was an obvious shift in the circular dichroism (CD) spectra of the miR‐193a‐3p upon Trp binding (Figure 1E). This shift is specific for miR‐193a‐3p as evident by the fact that miR‐193a‐5p and Phe which are also decreased in tumor patients did not cause a shift (Figure S1I, Supporting Information). It is reported that Trp binds RNA among the hydrophobic side chains to form a loop.^[^ ^9a,b^ ^]^ With in silico analysis, we found that miR‐193a‐3p contains a complementary sequence at its end (ACUGG matches UGACC) that forms a loop with Trp (Figure S1J, Supporting Information). Based on our hypothesis, we designed a mutation of miR‐193a‐3p on the supposed binding motif “ACUGG” (Figure S1J, Supporting Information). The CD assay shows that the mutation of miR‐193a‐3p (Figure S1I, Supporting Information) resulted in no observed shift.

Surface plasmon resonance (SPR) binding analysis methodology is an optical technique for detecting the interaction of two different molecules in which one is mobile, and one is fixed on a thin gold film.^[^ ^17^ ^]^ We used the SPR technology to determine whether Trp was physically binding to miR‐193a‐3p. Biotinylated miR‐193a‐3p was immobilized on a streptavidin sensor chip. Trp was used as the analyte at a high (200 µM), medium (20 µM) and low (2 µM) concentration and run over the chip. The results showed that miR‐193a‐3p binds to Trp at all three concentrations. Also, increased binding signals were observed with the higher concentrations of Trp (Figure 1F).

Isothermal titration calorimetry (ITC) is a quantitative technique that can determine the binding affinity of the interaction between two or more molecules in solution.^[^ ^18^ ^]^ The binding affinity of Trp to the miR‐193a‐3p was further determined with ITC and was ΔH = 1.92 kcal mol^−1^ and ΔS = 1.6 kcal mol^−1^ K^−1^, leading to an apparent dissociation constant Kd of 5.3 µM (Figure 1G; Figure S1K, Supporting Information). However, no significant changes of the peaks were found in Trp mixed with mutated miR‐193a‐3p in the ITC assay (Figure S1L, Supporting Information).

Finally, to probe the Trp interaction with miR‐193a‐3p, we performed 3D structure modeling prediction in silico. The miR‐193a‐3p sequence was submitted to the online HDOCK SERVER and analyzed by Chimera 1.16 software. Based on the top three fitting results of the model utilizing Docking Score ranges from −112.81 to −107.44, and ligand rmsd (Å) ranges from 12.54 to 45.78. We speculate that the nitrogen atom in the amino group of Trp plays a mechanical role for Trp binding to miR‐193a‐3p, with linkage conferred via a hydrogen bond (Figure 1H).

We also investigated whether the miR‐193a is present in the Trp complex and whether the level of miR‐193a expression is regulated by Trp in an in vivo mouse model. We accomplished this by using an established mouse liver colon cancer metastatic model where the spleen is injected with CT26‐GFP colon cancer cells using a protocol we described elsewhere.^[^ ^11^ ^]^ Mice received a Trp‐free diet for 3 days after intra‐splenic injection, followed by gavage with biotin labeled Trp (300 mg k^−1^g) 12 h prior to euthanasia. GFP^+^‐CT26 cells were sorted by FACS. Biotinylated Trp associated miR groups were isolated using streptavidin bead pulldown technology. The data generated from the RT‐qPCR indicated that miR‐193a‐3p in the Trp pulldown complex was significantly decreased, while miR‐193a‐5p remained unchanged (Figure 1I) in the CT26 cells isolated from the spleen on day 14 compared with day 0 (Figure 1I). In comparison to the spleen, CT26 cells isolated from 14‐day tumor bearing liver metastatic niches contained the lowest level of all six miRs analyzed, suggesting that the microenvironment and elapsed time affect the level of Trp associated miRs. Collectively, these data suggested that Trp can bind to miR‐193a‐3p in the Trp/miR complex isolated from in vitro cultured cells and in an in vivo mouse colon cancer model.

Interestingly, we also found that Trp play a critical role in modulating intracellularly miR‐193a‐3p concentration. In consistence, the miR‐193a‐3p level was significantly decreased in Trp‐free medium, while pri‐miR‐193a and miR‐193a‐5p level was not changed. Moreover, the concentration of miR‐193a‐3p was recovered when supplied the cells with Trp at 200 µM.

### 2.2 miRs in the Trp/miR Complex Work as a Group and Have a Stronger Activity on the Regulation of miR Targeted Gene Expression than Individual miRs

Since Trp can form a complex with miRs, as demonstrated in both an in vitro CT26 cell line and in an in vivo mouse model, we then determined whether the Trp centered miR complex contributes to the regulation of cellular biological activity. We performed a gene ontology (GO) analysis focusing on the signaling pathways. Kyoto Encyclopedia of Genes and Genomes (KEGG) and GO analysis indicate that this Trp centered miR complex is closely related to regulating the activity of multiple candidate genes via MAPK, and cancer‐related and the adherent junction signaling pathways. (Figure S2A, Supporting Information).

Next, we sought to experimentally demonstrate the role of a Trp‐miR complex using streptavidin pulldown strategy. We isolated biotin labeled Trp complexes from liver, LI and SI tissue of BALB/c mice given biotin labeled Trp (300 mg k^−1^g) by gavage. We then packed the isolated complexes in DOPE liposomes. The effects of miR and miR‐193a‐3p in the Trp complex were evaluated by western blot analysis and tumor cell growth. We previously demonstrated that miR‐193a‐3p acts as a tumor suppressor by inhibiting the expression of caprin1 and its downstream genes, including CCND2 and c‐Myc^[^ ^12^ ^]^ which play a critical role in cancer cell growth. Western blot data indicates that the cell growth related proteins c‐Myc and cyclin D2 expression were reduced in Trp complex treated cells. The inhibitory effect was reversed after Trp complex was pretreated with RNase or reduced after co‐transfecting cells with antisense‐miR‐193a‐3p (Figure S2B, Supporting Information). These results agreed with the fact that after transfecting CT26 cells for 36 h, we observed that Trp complexes inhibited tumor cell growth (Figure S2C, Supporting Information). Transfection with scrambled miR or Trp complex treated with RNase had no effect on tumor cell growth (Figure S2C, Supporting Information). The inhibitory effect was decreased when cells were co‐transfected with antisense‐miR‐193a‐3p (Figure S2C, Supporting Information). These results suggest that Trp‐miR‐193a‐3p plays a role in inhibiting tumor cell growth.

**Figure 2:**
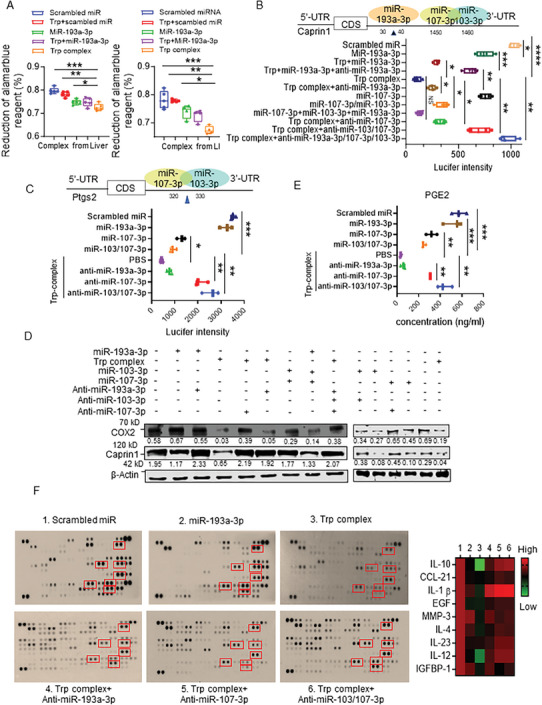
miRs in the Trp/miR complex work as a group and have a stronger activity on the regulation of miR targeted gene expression than individual miRs A). CT26 cells were transfected with scrambled miR (10 µM), Trp plus scrambled RNA, Trp‐miR‐193a‐3p (200 µM Trp plus 10 µM miR) or Trp complex (200 µM) derived from mouse liver (left panel) or LI tissue (right panel) for 24 h. Cell viabilities were assessed using the Cell‐Quant AlamarBlue Cell Viability Reagent. *p*‐values were calculated by means of an ANOVA test. ^*^ *p* < 0.05, ^**^ *p* < 0.01, ^***^ *p* < 0.001. B) Top panel, schematic of binding position of miR‐193a‐3p, miR‐103‐3p and miR‐107‐3p in caprin1 3′ untranslated regions (UTR). Bottom panel, luciferase activity assays of caprin1 3′UTR luciferase reporters after CT26 cells were co‐transfection with scrambled miR (10 µM), miR‐193a‐3p (10 µM), Trp (200 µM) or miR‐193a‐3p (10 µM), Trp compound plus antisense‐miR‐193a‐3p (10 µM), Trp complex (200 µM), miR‐107‐3p (10 µM), miR‐103‐3p/miR‐107‐3p, miR‐193a‐3p/103‐3p/107‐3p (10 µM for each miR), Trp complex plus antisense‐miR‐193a‐3p (10 µM), antisense‐miR‐103‐3p (10 µM) or antisense‐miR‐107‐3p (10 µM) and Trp complex plus antisense‐193a‐3p/103‐3p/107‐3p (10 µM for each antisense‐miR). The luciferase activity of each sample was determined using a reader containing a lucifer channel. P values were calculated by means of an ANOVA test. ^*^ *p* < 0.05, ^**^ *p* < 0.01, ^***^ *p* < 0.001 and ^****^ *p* < 0.0001. C). Top panel, schematic diagram of the putative binding sites of miR‐103‐3p/107‐3p in Ptgs2 (COX2) 3′‐UTR. Bottom panel, luciferase activity assays of Ptgs2 3′UTR luciferase reporters after co‐transfection with scrambled miR, Trp complex (200 µM)), Trp complex plus antisense‐miR‐103‐3p (10 µM) or 107‐3p (10 µM), miR‐103‐3p (10 µM), miR‐107‐3p (10 µM), miR‐103‐3p plus antisense‐miR‐103‐3p (10 µM) or miR‐107‐3p plus antisense‐miR‐107‐3p (10 µM). The luciferase activity of each sample was determined by a reader containing a lucifer channel. P values were calculated by means of ANOVA test. ^**^ *p* < 0.01, ^***^ *p* < 0.001. D). Total proteins were extracted from CT26 cells transfected for 24 h with scrambled miR (10 µM), miR‐193a‐3p (10 µM), miR‐193a‐3p (10 µM) plus antisense‐miR‐193a‐3p (10 µM), Trp complex, Trp complex plus antisense‐miR‐107‐3p (10 µM), Trp complex plus antisense‐miR‐193a‐3p (10 µM), Trp complex plus antisense‐miR‐107‐3p (10 µM) or antisense‐miR‐103‐3p (10 µM), miR‐193a‐3p (10 µM) mixed with miR‐103‐3p (10 µM) or miR‐107‐3p (10 µM), Trp complex plus antisense‐miR‐193a‐3p/103‐3p/107‐3p (10 µM for each miR), miR‐103‐3p (10 µM) plus antisense‐miR‐103‐3p (10 µM), miR‐103‐3p (10 µM) and miR‐107‐3p (10 µM) plus antisense‐miR‐107‐3p (10 µM), miR‐107‐3p (10 µM). Western blots of caprin1 and COX2 were performed, and band intensity was quantified and normalized to β‐actin. The results are presented between the panels (n = 3). E). ELISA of prostaglandin E2 (PGE2) concentration in cell medium was performed after co‐transfection with scrambled miR, Trp complex (200 µM), Trp complex plus antisense‐miR‐103‐3p (10 µM) and/or antisense‐miR‐107‐3p (10 µM), miR‐103‐3p (10 µM) and/or miR‐107‐3p (10 µM). The fluorescent signal of each sample was determined using a fluorescent reader (BioTek). P values were calculated by means of an ANOVA test. ^**^ *p* < 0.01, ^***^ *p* < 0.001. F). CT26 cells were transfected for 24 h with scrambled miR (10 µM), miR‐193a‐3p (10 µM), Trp complex (200 µM), Trp complex plus antisense‐miR‐193a‐3p, antisense‐miR‐103‐3p (10 µM) or antisense‐miR‐107‐3p (10 µM). Cell medium was collected and a cytokine array was performed (Left panel). The cytokine level was quantified and displayed as a heat map in the right panel.

We then tested whether miR‐193a‐3p in the Trp/miR complex has a stronger activity than miR‐193a‐3p alone or Trp plus miR‐193a‐3p which mimics an in vivo situation where free‐form Trp and miR‐193a‐3p could interact with the same recipient cell. CT26 cells were treated with miR‐193a‐3p alone or the Trp/miR complex derived from liver or LI tissue as described in FigureS2B (Supporting Information); Trp plus scrambled miR or Trp plus miR‐193a‐3p and scrambled miR were used as controls. Cell viability analysis results indicated that the Trp centered miR complex has superior inhibitory effects on cancer cell growth (**Figure** **2**A) in comparison with the results generated from cells treated with miR‐193a‐3p alone or Trp plus miR‐193a‐3p. Moreover, the Trp‐miR complex has a stronger inhibitory effect on CT26 tumor cell migration and invasion when assessed using wound healing and transwell assays when compared to miR‐193a‐3p alone or Trp plus miR‐193a‐3p (Figure S2D,E, Supporting Information). Collectively, these data indicate that Trp associated miRs working together have a stronger inhibitory effect on tumor cell growth, migration, and invasion than an individual miR such as miR‐193a‐3p and the free‐form of Trp plus miR‐193a‐3p. Based on these results, since the free form of Trp plus scramble miR had no effect on the phenotypes we analyzed, from this point forward experiments were conducted for the comparison of the biological effects of Trp‐miR complex with individual free‐form miR but not the free‐form of Trp.

Next, we determined the molecular mechanism underlying why miR‐193‐3p in the Trp/miR complex has a higher activity than miR‐193‐3p alone in terms of the phenotypes we analyzed above. We hypothesized that other miRs in the Trp/miR complex may target the same genes targeted by miR‐193‐3p leading to stronger activity. Since we found miR‐193a‐3p, miR‐103‐3p, and miR‐107‐3p in a Trp centered miR complex (Figure 1C), we searched three public miR databases (TargetScan, miRPathDB and MicroRNA) and found that miR‐193a‐3p and miR‐103‐3p/miR‐107‐3p may target the same mRNA 3′‐UTR of caprin1 mRNA on a conserved binding site from position of 41–49 and 1452–1460, respectively (Figure S2F, Supporting Information). To determine the role of Trp in miR‐103‐3p/miR‐107‐3p mediated targeting of caprin1 in colon cancer cells, we co‐transfected Trp/miR complex with luciferase sensor constructs containing the caprin1 3′ UTR cloned in the pEZX‐MT05‐Gluc vector (GeneCopoeia). Transfection of miR‐193a‐3p and miR‐107‐3p mimics alone or scramble miR served as controls (Figure 2B). Luciferase reporter assay results indicate that Trp/miR complex significantly reduced luciferase activity by nearly sixfold in comparison with miR‐193a‐3p mimics. Trp/miR complex also showed superior reduction in luciferase activity compared to co‐overexpression of miR‐193a‐3p and miR‐103‐3p/miR‐107‐3p. In addition, the enhancement of Trp/miR complex effects was alleviated by using antisense‐miR‐193a‐3p alone or together with antisense‐miR‐103‐3p/miR‐107‐3p (Figure 2B). These results suggested that miR‐193a‐3p/miR‐103‐3p/miR‐107‐3p in the Trp centered miR complex work together to create a stronger downstream biological effect on the caprin1 gene.

The conclusion that an miR, such as miR193a‐3p, in the Trp complex has better biological activity than the individual miR alone is also supported by the data generated from miR‐103‐3p and miR‐107‐3p in the Trp complex. miR‐103‐3p and miR‐107‐3p in the Trp complex target the ptgs2 gene, according to TargetScan, miRPathDB and MicroRNA search results. These databases predicted that miR‐103‐3p/miR‐107‐3p targets the ptgs2 (COX2) gene at the nucleic acid site of 323–330 (Figure S2F, Supporting Information). Then, we used the same luciferase reporter assay approach as we did for caprin1 and observed that the Trp/miR complex more effectively suppressed luciferase activity than miR‐103‐3p or miR‐107‐3p alone or together. Pre‐treatment of the Trp complex with antisense‐miR‐103‐3p/miR‐107‐3p abolished the inhibitory effects of the Trp/miR complex (Figure 2C). The results generated from the luciferase reporter assay also agreed with the western blot analysis indicating that Trp complex treatment leads to a stronger reduction of expression of caprin1 and COX2 than miR‐193a‐3p, miR‐103‐3p, and miR‐107‐3p mimics alone or together (Figure 2D). However, the data generated from triple (miR‐107‐3p + miR‐103‐3p + miR‐193a‐3p) treatment (Figure 2F) agreed with the results from RT‐qPCR and western blot that the level of caprin1 or COX2 in CT26 cells treated with Trp complex or triple miRs was not statistically different (Figure S2G,H, Supporting Information). This suggested that Trp complex inhibition of caprin1 or COX2 is mainly dependent on the effects of the triple miRs (miR‐193a‐3p, miR‐103‐3p, and miR‐107‐3p).

Pre‐treatment of the Trp/miR complex with antisense‐miR‐103‐3p/miR‐107‐3p reversed COX2 inhibition (Figure 2D). The COX2‐PGE2 pathway plays a critical role in the induction of inflammatory cytokines that contribute to tumor progression via multiple mechanisms.^[^ ^19^ ^]^ We observed much lower PGE2 concentrations in the medium of CT26 cells after incubating them with the Trp complex, compared to scrambled miR, miR‐193a‐3p and miR‐103‐3p/miR‐107‐3p mimic treatments alone. The Trp complex‐mediated inhibition of PGE2 release was reversed by co‐transfecting the Trp complex with antisense‐miR‐103‐3p/miR‐107‐3p (Figure 2E). Cytokine array analysis results further indicated that cytokine levels of IL‐10, IL‐4, MMP‐3, CCL‐21, IL‐12, and IGFBP‐1 were reduced in the Trp complex treated CT26 cell medium compared to cells treated with scrambled miR and miR‐193a‐3p alone (Figure 2F; Figure S2I, Supporting Information). Similarly, the inhibitory effect of Trp complex was significantly reversed by antisense‐miR‐103‐3p and antisense‐miR‐103‐3p/miR‐107‐3p (Figure 2F). ELISA results confirmed the cytokine array data that Trp complex treatment significantly decreased levels of interleukin IL‐4, IL‐1β, IL‐10 and IL‐12 (Figure S2J, Supporting Information).

Collectively, these data support the hypothesis that miRs such as miR‐193a‐3p, miR‐103‐3p, and miR‐107‐3p in the Trp/miR complex work as a group and have a stronger activity than individual miR to inhibit expression of miR targeted genes, such as caprin1 and COX2.

### 2.3 Trp Promotes miR‐193a‐Mediated Targeted mRNA Cleavage by Enhancing Ago2 RNase Activity

Mature miR, which enters the RNA‐induced silencing complexes (RISC), is capable of binding to partially complementary sequences within the 3′UTR of the target mRNA.^[^ ^20^ ^]^ Ago2 has been described as a RISC slicer, provided that the RNase cleavage activity targets mRNAs that complement the guiding miR. Ago2 is the only protein component required for RISC activity.^[^ ^6^ ^]^ Ago2 PIWI domain possesses a Trp binding region, composed of three Trp‐binding pockets. This domain allows Ago2 to interact with diverse unstructured glycine/tryptophan (GW)‐rich proteins in RISC.^[^ ^7^ ^]^ Interactions between Ago2 and GW‐rich TNRC6B in RISC promote the assembly of massive complexes and control of Ago2 RNase activity.^[^ ^7b,c^ ^]^

**Figure 3:**
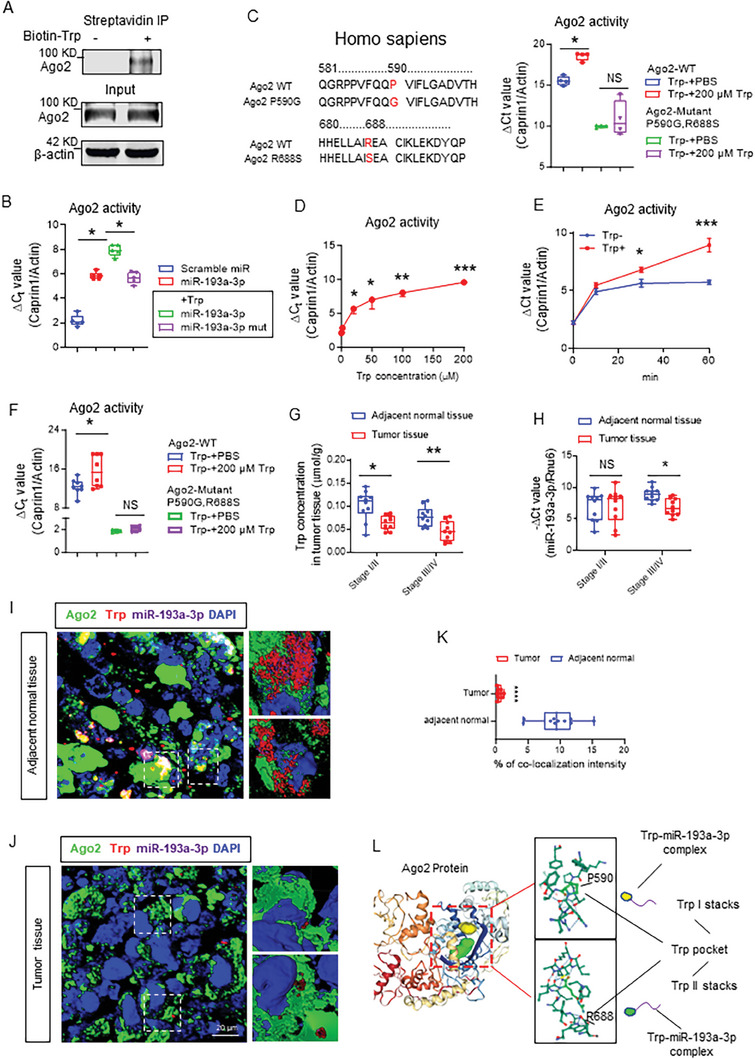
Trp promotes miR‐193a‐mediated targeted mRNA cleavage by enhancing Ago2 RNase activity. A). SW620 cells were treated with or without biotinylated Trp (200 µM) for 12 h in Trp depletion medium. Biotin‐Trp was pulled down with streptavidin beads and Ago2 protein in the pulldown was analyzed by western blot. B). An in vitro Ago2 activity assay was performed using Ago2 and miR‐193a‐3p (500 nM) or miR‐193a‐3p mutant (mut, 500 nM) in the presence of Trp (200 µM), followed by adding 2.5 µg mRNA extracted from SW620 cells. Expression of caprin1 was determined by RT‐qPCR. P values were calculated by means of an ANOVA test. ^*^ *p* < 0.05. C). An in vitro Ago2 activity assay was performed using Ago2 wild‐type (WT) or mutant (mut) recombinant protein with miR‐193a‐3p in the presence of Trp (200 µM) or in Trp deletion medium, followed by adding 2.5 µg mRNA from SW620 cells. Expression of caprin1 was determined by RT‐qPCR. P values were calculated by means of an ANOVA test. ^*^ *p* < 0.05, NS, no significance. D). An in vitro Ago2 activity assay was performed using Ago2 and guide miR‐193a‐3p in the presence of Trp at the indicated dose, followed by adding mRNA from SW620 cells. Expression of caprin1 was determined by RT‐qPCR. P values were calculated by means of an ANOVA test. ^*^ *p* < 0.05, ^**^ *p*<0.01, ^***^ *p* < 0.001. E). An in vitro Ago2 activity assay was performed using Ago2 and guide miR‐193a‐3p or Trp‐miR‐193a‐3p, followed by adding mRNA from SW620 cells. The reaction was stopped at the indicated time point. Expression of caprin1 was determined by RT‐qPCR. P values were calculated by means of an ANOVA test. ^*^ *p* < 0.05, ^***^ *p* < 0.001. F). SW620 were transfected with Flag‐Ago2‐WT or Flag‐Ago2 mutant (P590G and R688S) plasmid for 48 h. Transfected SW620 cells were cultured in Trp depleted medium for 12 h, and then treated with Trp (200 µM) or PBS as a control for culturing additional 12 h before the cells were harvested. Ago2 complex was pulled down using protein G beads cross‐linked with anti‐Flag M2 antibody (Sigma–Aldrich) and eluted by 3xFlag peptide (Rockland Immunochemicals, PA). An Ago2 activity assay was performed using Ago2 complex as mentioned and guide miR‐193a‐3p in the presence of Trp, followed by adding mRNA from SW620 cells. The reaction was stopped at the indicated time point. Expression of caprin1 was determined by RT‐qPCR. P values were calculated by means of an ANOVA test. ^*^ *p* < 0.05, NS, no significance. G). Trp concentration in the adjacent normal tissue or in the tumor tissue of patients in cancer stage I/II (circles) and stage III/IV (squares) were determined by HPLC. ^*^ *p* < 0.05, ^**^ *p* < 0.01. H). MiR‐193a‐3p level in the adjacent normal tissue or in the tumor tissue of patients in cancer stage I/II (circles) and stage III/IV (squares) were determined by RT‐qPCR. ^*^ *p* < 0.05, ^**^ *p* < 0.01. I,J). Fluorescence in situ hybridization of tumor sections from human colon samples stained with biotinylated Ago2 (green) were overlayed with Trp (red) and miR‐193a‐3p (purple). Five random fields were photographed in z‐stack and representative 3D reconstruction images were obtained using “surface” plug‐in in Imaris software. Scale bars, 40 µm. K) The percentage of co‐localization intensity of Ago2, Trp and miR‐193a‐3p were analyzed by Image J software and the results is shown. L). Cartoon figure shows the structure of the Ago2 protein containing a tandem tryptophan pocket bound to Trp‐miR‐193a‐3p complex.

To further demonstrate whether the Ago2 is involved in Trp/miR‐193a‐3p mediated inhibition of CT26 tumor cell growth, the Ago2 inhibitor BCI‐137 (Sigma) was used to pretreat the CT26 cells. BCI‐137 inhibits binding of miR to Ago2. We found that Trp complex isolated from CT26 cells pretreated with BCI‐137 does not inhibit CT26 growth, suggesting that Ago2 is indeed involved in the Trp complex mediated inhibition of CT26 tumor cell growth (Figure S3A, Supporting Information). Therefore, we hypothesize that Trp in the Trp/miR complex described in this paper may interact with Ago2, and subsequently enhances Ago2 RNase activity. We used miR‐193a‐3p as a proof‐of‐concept. We first determined whether Ago2 is present in the Trp/miR‐193a‐3p complex. Western blot analysis for the presence of Ago2 in the Trp complex indicated that Ago2 can be detected by streptavidin pulldown of biotin labeled Trp complex isolated from the human colon cancer cell line SW620 (**Figure** **3**A), suggesting that Ago2 is present in the Trp complex isolated from tumor cells. Next, we determined whether Ago2 in the Trp complex has RNase activity. We began by establishing the protocol for measuring Ago2 activity in vitro using a previously published protocol.^[^ ^21^ ^]^ Recombinant Ago2 or bovine serum albumin (BSA) control was preincubated with miR‐193a‐3p, followed by the addition of a mixture of total mRNA extracted from SW620 cells. After incubation in a 37 °C water bath for 1 h, the cleavage of caprin1 mRNA which is targeted by miR‐193a‐3p was assessed by RT‐qPCR using specific PCR primers that can amplify the caprin1 coding regions (2129 bp in full length), including nucleotide sequences at positions 161 to 487, 487 to 1366, and 1366 to 1959. The RT‐qPCR results indicate that Ago2 cleavage occurs between nucleotide sequences 487 and 1366 bp (Figure S3B, Supporting Information); thus, we used this cleavage product to assess Ago2 RNase activity.

The addition of Trp increased the efficiency of caprin1 gene suppression by up to fourfold compared to miR‐193a‐3p + Ago2 alone (Figure 3B), suggesting that Trp promotes Ago2 mediated inhibition of caprin1 gene expression. The specificity of cleavage is supported by the fact that the addition of mutant miR‐193a‐3p did not alter caprin1 gene expression (Figure 3B). Furthermore, Ago2 possessing mutant Trp binding sites (P560G and R688S)^[^ ^7c^ ^]^ also failed to mediate inhibition of caprin1 gene expression (Figure 3C). Collectively, these results demonstrate that both Trp and Trp binding sites in the Ago2 are required to enhance miR‐193a‐3p‐mediated inhibition of caprin1 targeted gene expression. This finding is significant since increased Ago2 RNase activity not only enhances miR‐193a‐3p activity, but also enhances other miRs as well.^[^ ^7c^ ^]^

Our data show that blood levels of Trp are lower in colon cancer patients than in healthy subjects (Figure 1A). Whether the level of Trp affects Ago2 mediated degradation of miR targeted mRNA is not known. Using the same assay as above, we showed that while holding Ago2 levels constant in the reaction, reduction of gene expression of caprin1 in human SW620 (Figure 3D) or mouse CT26 cells (Figure S3C, Supporting Information) occurs by a Trp dose‐dependent mechanism. This result generated in test tube form was reproduced in live cells. The expression of caprin1 in SW620 cells was decreased in the presence of Trp and the RT‐qPCR results further indicate that Ago2 mediated cleavage of caprin1 (between nucleotide sequences 487 and 1366 bp) was attenuated (Figure S3D, Supporting Information) when the cells were cultured with Trp deficient cell culture media (Figure S3D, Supporting Information). Moreover, time‐dependent effects on Trp mediated enhancements of caprin1 slicing were further demonstrated (Figure 3E).

Next, the data generated from test tube reactions were further confirmed in live cells, using FLAG‐tagged Ago2 wild‐type (WT) and mutant Ago2 (P590G and R688S) complexes pulled down from SW620 and CT26 cells treated with or without Trp. We observed that Ago2 complex isolated from Trp treated SW620 (Figure 3F) and CT26 cells (Figure S3E, Supporting Information) more robustly inhibits caprin1 expression compared to PBS treated cells. This enhancement was cancelled by using Ago2 mutants P590G and R688S in testing (Figure 3F; Figure S3E, Supporting Information).

To further relate the levels of Trp and miR‐193a‐3p expression in human colon cancer tissue to disease progression, twenty patients with stage I to IV colon cancer were examined. miR‐193a‐3p expression or plasma/colonic tissue levels of Trp were quantitatively analyzed using RT‐qPCR or HPLC, respectively. Although no significant changes in plasma Trp concentrations were observed between the cohorts of cancer stages I/II and stages III/IV (Figure S3F, Supporting Information), Trp concentrations were lower in tumor tissue compared to adjacent normal tissue of patients in all stages of colon cancer (Figure 3G). The downregulation of miR‐193a‐3p expression in tumor tissue was observed in late stages (stage III/IV) but not early stages (stage I/II) of colon cancer patients (Figure 3H).

We then determined whether the Trp centered miR complex we discovered in the colon cancer cells was present in human colon tissue and mouse metastatic liver tissue. We performed miR‐193a‐3p fluorescence in situ hybridization (FISH) and co‐stained with Trp using specific anti‐Trp antibody in tumor tissue sections. Confocal immune staining results indicated that intensity signals correlated with lesser numbers of double positive miR‐193a‐3p/Trp in human colon tumor tissue (Figure 3I,J) and mouse liver metastases compared to adjacent normal tissue (Figure S3G, Supporting Information). Since Ago2 is recruited into the Trp/miR complex in the CT26 cells, we further tested whether Ago2 is colocalized with Trp in human colon tumor tissue and mouse metastatic liver tissue. Confocal immune staining indicates that Ago2 co‐localizes with Trp, and that a much stronger interaction signal is detected in adjacent normal tissue than in tumor tissue (Figure 3I–K; Figure S3H, Supporting Information). To summarize, our data suggests that Trp enhances Ago2 RNase activity by interacting with Ago2 Trp‐binding pockets (Figure 3L).

Since caprin1 and COX2 expression is inhibited by the Trp/miR complex, we measured expression levels of both caprin1 and COX2 in the relevant tissues. Immunostaining indicates that protein expression of caprin1 and COX2 is much stronger in colon tumor tissue than in adjacent normal tissue (Figure S3I, Supporting Information). This result is also supported by the fact that expression of miR‐103‐3p/miR‐107‐3p, which inhibits expression of both genes encoding caprin1 and COX2, is lower in patients with advanced tumor stages III/IV than with early tumor stages I/II (Figure S3I, Supporting Information). Our results are also supported by data analysis of the TCGA database. Results from TCGA analysis indicate that the expression of caprin1 (Figure S3J, Supporting Information) and COX2 (Figure S3K, Supporting Information) is higher in the colon tumor tissue (CRC) than in normal tissues. TCGA data and miR profiling from dataset GSE73487 also indicates that the level of hsa‐miR‐193a (Figure S3L, Supporting Information) hsa‐miR‐107 (Figure S3M, Supporting Information) was lower in CRC than in normal tissue.

### 2.4 Trp Organized miR‐193a‐3p/miR‐103‐3p/miR‐107‐3p Works as a Group to Inhibit Colon Cancer Metastasis to the Liver

**Figure 4:**
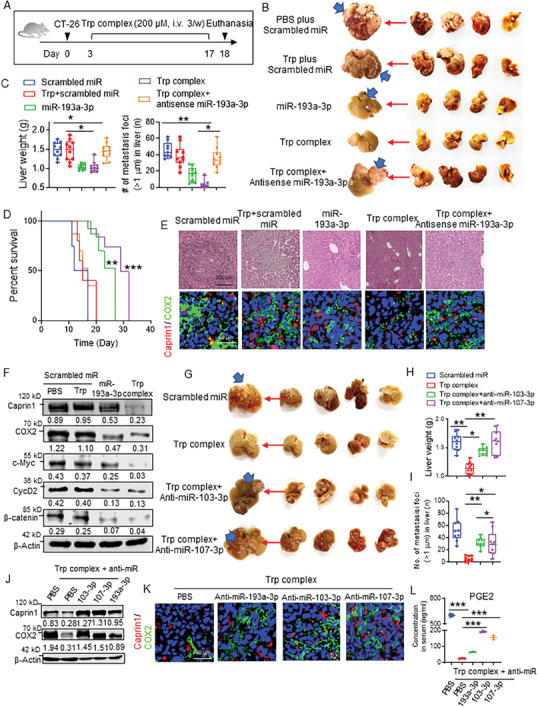
Trp/miR‐193a‐3p/miR‐103‐3p/miR‐107‐3p works as a unit to inhibit colon cancer metastasis to the liver. A). Protocol used in BALB/c mice for intrasplenic injection of CT26 cells as well as Trp and miR treatment. B). Representative livers (metastatic nodules indicated by blue arrows) from 18‐day tumor‐bearing BALB/c mice (n = 10) *i.v*. injected with PBS plus scrambled miR, Trp (200 µM) plus scrambled miR, PBS plus miR‐193a‐3p, Trp complex (200 µM) or Trp complex plus antisense‐miR‐193a‐3p (10 µM). C). Liver weight (right, top panel) and number of metastatic foci in liver were quantitatively analyzed. P values were calculated by means of an ANOVA test. ^*^ *p* < 0.05 and ^**^ *p* < 0.01. D). Survival analysis of BALB/c mice after intrasplenic injection of CT26 cells treated as indicated (n = 10 per group). *p*‐values were calculated by means of an ANOVA test. ^**^ *p* < 0.01 and ^***^ *p* < 0.001. E). H&E‐stained sections of tumor‐bearing livers (scale bar 100 µm, top; scale bar 40 µm, bottom) from mice treated with PBS plus scrambled miR, Trp (200 µM) plus scrambled miR, PBS plus miR‐193a‐3p, Trp complex (200 µM) or Trp complex plus antisense‐miR‐193a‐3p (10 µM). Fluorescence images of tumor sections stained with COX2 (green) were overlayed on caprin1 (red) and DAPI (blue). Five random fields were photographed, and representative images are shown. Scale bars, 40 µm. F). Western blot analysis showing the level of miR‐193a‐3p target protein of caprin1, COX2, c‐Myc, β‐catenin and cyclinD2 in liver cancer tissue from mice treated with PBS plus scrambled miR, Trp plus scrambled miR, PBS plus miR‐193a‐3p or Trp binding miR‐193a‐3p complex. Band intensities are normalized to β‐actin and the results are presented between the panels. G). Representative livers (metastatic nodules indicated by blue arrows) from 18‐day tumor‐bearing BALB/c mice (n = 10) *i.v*. injected with PBS plus scrambled miR, Trp complex (200 µM) and Trp complex plus antisense‐miR‐103‐3p or antisense‐miR‐107‐3p (10 µM). H). Liver tissue from 18‐day tumor‐bearing BALB/c mice (n = 10) *i.v*. injected with PBS plus scrambled miR, Trp complex (200 µM) and Trp complex plus antisense‐miR‐103‐3p or antisense‐miR‐107‐3p (10 µM) were collected. Liver weights were quantitatively analyzed. *p*‐values were calculated by means of an ANOVA test. ^*^ *p* < 0.05 and ^**^ *p* < 0.01. I). Liver tissue from 18‐day tumor‐bearing BALB/c mice (n = 10) *i.v*. injected with PBS plus scrambled miR, Trp complex (200 µM) and Trp complex plus antisense‐miR‐103‐3p or antisense‐miR‐107‐3p (10 µM) were collected. Number of metastatic foci in liver were quantitatively analyzed. *p*‐values were calculated by means of an ANOVA test. ^*^ *p* < 0.05 and ^**^ *p* < 0.01. J). Western blot analysis showing the level of miR‐193a‐3p target protein of caprin1 and COX2 in liver cancer tissue from mice *i.v*. injected with scrambled miR, Trp complex (200 µM) and Trp complex plus antisense‐miR‐103‐3p or antisense‐miR‐107‐3p (10 µM). Band intensities are normalized to β‐actin and the results are presented between the panels. K). Fluorescence images of tumor sections from mice treated with PBS plus scrambled miR, Trp complex, Trp complex plus antisense‐miR‐103‐3p or Trp complex plus antisense‐miR‐107‐3p (10 µM) stained with COX2 (green) and overlayed with caprin1 (red) and DAPI (blue). Five random fields were photographed, and representative images are shown. Scale bars, 40 µm. L). ELISAs of PGE2 concentration in serum from 18‐day tumor bearing mice were performed after mice were *i.v*. injected with scrambled miR, Trp complex (200 µM)), Trp complex plus antisense‐miR‐103‐3p (10 µM) and/or miR‐103‐3p (10 µM), miR‐107‐3p (10 µM) and/or miR‐107‐3p (10 µM). The fluorescent signal of each sample was determined using a fluorescent reader. *p*‐values were calculated by means of an ANOVA test. ^***^ *p* < 0.001.

Using an in vitro assay, we demonstrated that Trp/miR‐193a‐3p/miR‐103‐3p/miR‐107‐3p complex plays a crucial role in inhibiting CT26 cell growth and migration (Figure S2B–E, Supporting Information). Next, we tested whether tumor metastasis and growth inhibition can be observed in the liver in a mouse colon cancer model (**Figure** **4**A). All CT26 tumor carrying mice were fed a Trp depleted diet and were randomly divided into five groups and treated with liposomes described as follows: Trp complex, Trp complex plus antisense‐miR‐193a‐3p, miR‐193a‐3p mimic alone, Trp plus scramble miR or PBS, as a control group. All treatment modalities were incorporated into cationic liposomes made of DOTAP/DOPE. Liposomes were injected intravenously into the 3‐day tumor bearing mice 3 times per week for 2 weeks. After the last treatment, mice were euthanized; numbers and size of liver tumor metastases were counted. Intravenous injection of liposomes packed with the Trp complex increased the level of miR‐193a and Trp detected in CT26 liver metastases, while the level of exosomal miR‐193a‐3p was reduced (Figure S4A,B, Supporting Information). Tallies indicate that treatment with Trp complex containing liposomes resulted in lower numbers and smaller sized liver tumors compared with animals treated with miR‐193a‐3p alone, Trp‐scrambled miR complex or scrambled miR (Figure 4B,C). Trp complex plus antisense miR‐193a‐3p reversed the inhibitory effect of Trp complex on metastasis of tumor cells to the liver. Also, mice treated with Trp complex experienced delayed metastasis of tumor cells to the liver and increased survival of tumor bearing mice compared to mice treated with miR‐193a‐3p alone, Trp‐scrambled miR complex or PBS treatment (Figure 4D,E). The western blot results show that Trp complex decreased caprin1, COX2, cyclinD2, c‐Myc, and β‐catenin expression (Figure 4F).

We further tested whether miR‐103‐3p and miR‐107‐3p in the Trp/miR complex are also involved in reduction of tumor progression. We used the same colon cancer metastatic liver model as described by Teng.^[^ ^12^ ^]^ Three days after initial injection of CT26 cells into the spleen, mice were injected intravenously with liposomes containing the Trp complex and antisense miR‐103‐3p or miR‐107‐3p. Our results indicated that antisense‐miR‐103‐3p and antisense‐miR‐107‐3p abrogated the inhibitory effects of Trp complex on tumor development (Figure 4G). Liver weight and tumor numbers were significantly increased in mice treated with Trp complex plus antisense‐miR‐103‐3p or antisense‐miR‐107‐3p compared to Trp complex treatment alone (Figure 4H,I). Western blot and immunobiological staining indicate that treatment with Trp complex inhibited caprin1 and COX2 expression and adding antisense‐miR‐103‐3p or antisense‐miR‐107‐3p to Trp complex cancelled Trp complex‐mediated inhibitory effects on tumor cell metastasis to the liver (Figure 4J,K). Moreover, tumor bearing mice injected with Trp plus antisense‐miR‐103‐3p/miR‐107‐3p have higher serum concentrations of PGE2 compared to mice administered Trp complex alone (Figure 4L).

We further tested whether the Trp associated miR‐103‐3p and miR‐107‐3p inhibition of tumor progression depended on COX2. We transfected CT26 cells to overexpress COX2 using Crispr/Cas9 lentivirus particles. After puromycin selection, we selected CT26 cell lines stably overexpressing COX2 (Figure S4C, Supporting Information). Mice then underwent splenic injections of COX2 overexpressing CT26 cells to establish a colon cancer liver metastasis model. Mice were administered Trp complex intravenously or RNase treated Trp complex, or Trp complex plus antisense‐miR‐193a‐3p, antisense‐miR‐193a‐3p/107‐3p/103‐3p, antisense‐miR‐107‐3p and Trp complex plus antisense‐miR‐103‐3p. Treatment with Trp complex reduced liver weight and the number of tumor foci, while RNase treatment of Trp complex did nothing to reduce liver weight or tumor load (Figure S4D–G, Supporting Information). In addition, antisense‐miR‐103‐3p/107‐3p undermined the effects of Trp complex on tumor progression inhibition (Figure S4D–G, Supporting Information), for the same reason that miR‐103‐3p and miR‐107‐3p inhibiting the expression of COX2 in liver (Figure S4H, Supporting Information). Collectively, Trp centered miR‐193a‐3p/miR‐103‐3p/107‐3p must work in concert to inhibit metastasis of colon cancer cells to the liver by targeting the effects of caprin1 and COX2 gene products.

## 3 Discussion

The data published in many labs have shown that the level of tryptophan regulates many pathways.^[^ ^22^ ^]^ The results presented in this study point to a unified assembly model explaining how an essential amino acid like tryptophan communicates with a miR, such as miR‐193a, to regulate miR activity (Table of Contents). Collectively, we provide mechanistic insight into how tryptophan regulates miR activity by enhancing Ago2 RNase activity, which is a central hypothesis tested in this study. Our finding that tryptophan regulates miR activity by enhancing Ago2 RNase activity is significant because Trp is an essential amino acid. miRs and Ago2 play a ubiquitous role in gene regulation across all cells. Essential amino acids, miR, and Ago2 are involved in many physiological and pathophysiological processes, such as cellular differentiation, proliferation, apoptosis and development. Their dysregulation has been related to various pathological disorders, including cancer as demonstrated in this study. Specifically, what is presented as a proof‐of‐concept example is how tryptophan interacts with miR‐193a‐3p, miR‐103, and miR‐107 to inhibit colon cancer liver metastasis. The assembled Trp/miR complex has at least two major biological effects as demonstrated in this study. 1. Via interaction with Ago2, Trp enhances Ago2 RNase activity for miR mediated inhibition of miR targeted gene expression. 2. By aligning multiple miR in the Trp complex targeting the same gene, inhibition of expression of the targeted gene is enhanced.

Numerous studies have demonstrated that amino acids and miRs play crucial roles in virtually all cellular processes, regardless of whether the plant or animal kingdom is considered. Amino acids are fundamental molecules that not only serve as the building blocks for proteins but can also play a critical role in regulation of cell signaling pathways.^[^ ^23^ ^]^ In the human genome miRs are capable of modulating up to 60% of the protein‐coding genes at the translational level.^[^ ^10a^ ^]^ Ago2 serves as the catalytic engine of the RNA induced silencing complex and plays an important role in small RNAs, including miR guided post‐transcriptional gene silencing, through mRNA degradation^[^ ^24^ ^]^ and translational repression.^[^ ^25^ ^]^ Whether amino acids communicate with the miR community to regulate host cell biological activity was previously unknown.

In this study, we discovered that Trp serves as an organizing molecule that can bind to miR as well as Ago2‐Trp bound pockets and regulate miR activity by enhancing Ago2 RNase activity. This finding is significant because Trp is an essential amino acid and Ago2 plays a ubiquitous role in gene regulation across all cells. Essential amino acids, miR, and Ago2 are involved in many physiological and pathophysiological processes, such as cellular differentiation, proliferation, apoptosis and development, while their dysregulation has been related to various pathological disorders, including cancer^[^ ^26^ ^]^ and metastasis of colon cancer to the liver, as demonstrated in this study. As a proof‐of‐concept, our data presented in this study focused on the effect of Trp on miR‐193a activity in the context of a Trp/miR complex. However, we cannot exclude possible effects of Trp on the activity of other miRs in the Trp/miR complex, which will prompt further investigations to study the effects of Trp on the activity of other miRs. Also, in the Trp/miR complex, there are several miRs such as miR‐145a‐5p, miR‐106a‐5p and miR‐17‐5p that regulate the expression of oncogenes to promote/suppress the metastasis of cancer. Therefore, the finding that the expression of oncogene caprin1 is inhibited by miR‐193a opens a new avenue to further investigate whether other miRs that are recruited in the Trp/miR complex have an effect on the modulation of expression of their targeted oncogenes. Collectively, our study provides the foundation for future investigations of whether Trp or other essential amino acids can regulate global miR expression and activity in general.

Synergistic effects of miRs have been shown to be important for biological processes, such as neurogenesis^[^ ^27^ ^]^ and human embryonic stem cell pluripotency and differentiation.^[^ ^28^ ^]^ Highly synergistic miR regulation of target mRNA may also have implications in diseases, such as oncogenesis. For example, cyclin‐dependent kinase inhibitor 1A (CDKN1A), a tumor suppressor that is downregulated in multiple cancers,^[^ ^29^ ^]^ is targeted by at least 28 miRs and many of which are upregulated together in cancers where CDKN1A has been implicated.^[^ ^30^ ^]^ We found that the Trp centered miR complex works better than any individual miR on the inhibition of expression of targeted genes. The minimally miR‐induced silencing complex (miRISC) consists of a guide strand of matured miR and Ago2.^[^ ^31^ ^]^ The target specificity of miRISC is due to its interaction with complementary sequences on target mRNA, called miR response elements (MREs). An individual mRNA may contain many MREs^[^ ^32^ ^]^ and thus have different and multiple copies of miRs loaded on the same mRNA, such as miR‐193a‐3p/miR‐103‐3p/miR‐107‐3p as demonstrated in this study. Multiple MREs can act to increase miRISC occupancy. Cooperation of multiple, proximal MREs on target mRNA has been shown to increase retention times of miRISC complexes on those mRNAs, most likely through the three Ago2 Trp binding sites on the GW182 family proteins.^[^ ^7^, ^33^ ^]^ In our study, the formation of the Trp centered miR complex allows low expression, but functional miR selectively loaded on the miRISC complexes for synergistically increasing multiple miR activity on the same targeted mRNA. This finding provides a rationale for future investigations as to whether additional factors are required for Trp/miR complex mediated enhancing of the inhibition of expression of a targeted gene.

Trp is most likely lower in the tumor because indoleamine 2,3‐dioxygenase 1 (IDO1) which is overexpressed in many different types of tumor cells causes immunosuppression via the Trp metabolic pathway.^[^ ^15^, ^34^ ^]^ We also demonstrated that under pathological conditions, such as colon cancer, we can use therapeutic doses of Trp to treat tumor cells via intratumoral injection. As a result of intratumoral injection of Trp, indoleamine 2,3‐dioxygenase 1 (IDO1) which is overexpressed in many different type of tumor cells and causes immunosuppression via Trp metabolic pathway^[^ ^35^ ^]^ will be exhausted, leading to accumulating Trp and the Trp/miR complex as we demonstrated in this study.

In addition, in this study, although we have discovered the role of Trp/miR complex in the inhibition of colon cancer liver metastasis in a mouse model, whether the composition and biological activity of the Trp/miR complex isolated from colon tumor cells is different from the Trp/miR complex isolated from healthy colon tissue derived cells is not known. We are having difficulty in establishing a normal colon epithelial cell line to address this concern. But we predict the composition of the Trp/miR complex will be altered as the miR profile is altered under various pathological conditions when compared with physiological conditions.

Inflammation regulated by cytokines and chemokines has been demonstrated to be closely associated with all stages of development and malignant progression of most types of cancer. In this study, we demonstrated that the Trp/miR complex plays a role in regulating cytokine and chemokine expression. The expression of these cytokines and chemokines may not be directly regulated by miRs in the Trp/miR complex. Based on the data presented in this study, we show that miR‐103‐3p and miR‐107‐3p inhibit the expression of the COX2 gene which plays key roles in the hallmarks of cancer and alters the tumor microenvironment where these inflammatory cytokines could be induced. Therefore, our finding provides a rationale for future investigations as to whether miRs such as miR193a working with other miRs as a group in the context of the Trp/miR complex inhibit the expression of inflammation associated genes via directly targeting these inflammation genes, as well as targeting genes that regulate the pathways that regulate the expression of these inflammatory genes.

Our results presented in this study also indicate that Trp containing regular chow diet alters the expression of the miR profile compared with the miR profile generated from mice fed with a Trp deficient diet. These altered miRs do not necessarily interact with Trp. The expression of these miRs could be regulated via Trp derived metabolites that can modulate the activity of multiple pathways.^[^ ^23^, ^36^ ^]^ Therefore, our findings that Trp regulates the expression of miRs opens an avenue to further study which of the Trp mediated pathways regulate the expression of miR expression and their biology effect(s).

## 4 Experimental Section

### Animals

BALB/c and C57BL/6, 6‐to 10‐week‐old male mice, were purchased from Jackson Laboratory (Bar Harbor, ME). IDO‐1 KO (B6.129‐Ido1^1Alm^/J) mice was purchased from Jackson lab. A targeting vector was designed to replace exons 3–5 (encode critical portions of the IDO‐1 enzyme catalytic site) with the beta‐galactosidase and neomycin resistance genes. The construct was electroporated into 129/SvJ‐derived embryonic stem (ES) cells and correctly targeted clones were injected into blastocysts. Male chimeric mice were mated with C57BL/6 females to produce heterozygotes. Mutant mice were backcrossed more than 10 generations to C57BL/6 before arriving at The Jackson Laboratory. Mice were housed under specific‐pathogen‐free (SPF) conditions. Animal care was performed according to the Institute for Laboratory Animal Research (ILAR) regulations, and all animal procedures were approved by the University of Louisville Institutional Animal Care and Use Committee (Louisville, KY). Mice were provided with irradiated tryptophan (Trp)‐modified synthetic chows (Research Diets Inc., A11022501‐03, NJ, USA) or paired regular chow (Research Diets Inc., A11112201, NJ, USA) diet ad libitum for the indicated periods of time. The difference in the modified and paired diets was only the Trp content, with 0 w/w versus 0.3% w/w in the Trp and paired diets, respectively.

To generate animal models of metastatic colon cancer in the liver, 8‐ to 12‐week‐old male BALB/c mice (n = 10 per group) were anaesthetized with a mixture of ketamine and xylazine administered by intraperitoneal injection. Then 1×10^6^ CT26 colon cancer cells were administered via intrasplenic injection following a left subcostal incision through the skin and the peritoneum that exposed the spleen. After 3 days, all the mice were fed the Trp‐free diet. After treatment for 16 days, mice were killed, and livers or tumors were removed for examination.

### Trp Complex Pull Down

CT26 cells were grown as monolayers in 100 mm^2^ dishes until 80% confluency was achieved. The cells were then Trp starved overnight in Trp‐free medium (Cat#: D9807‐04, Life science). CT26 cells were treated with synthetized biotin labeled Trp (200 µM, ANASPEC, CA, USA) for 12 h. The cells were lysed in Qiazol lysis reagent (Qiagen). Total miR was extracted using a miRNeasy kit (Qiagen). Magnet Dynabead streptavidin conjugated beads (Thermo Fisher, M‐270, 100 µl) were added to each sample to pull down the biotin labeled Trp‐complex. After washing in washing buffer (10 mM Tris, 2 mM EDTA, 400 mM NaCl and 0.02% tween‐20), the immunocomplex was eluted either by elusion buffer ((biotin, 4 mg ml^−1^) in 25 mM Tris‐HCl containing 0.3 M NaCl (pH 8.5, as the elution buffer) at room temperature for 5 min or by Qiazol lysis buffer for 10 min with frequent agitation. miR associated to Trp complex was purified using a miRNeasy column. RT‐qPCR was performed to quantify miR.

BALB/c mice were fed the Trp‐free diet for 3 days after intra‐splenic injection and then gavage given biotin labeled Trp (300 mg k^−1^g) for 12 h before being euthanized. Tissues were homogenized in Qiazol lysis reagent (Qiagen). Total miR was extracted using a miRNeasy kit (Qiagen). Magnet Dynabead streptavidin conjugated beads (Thermo Fisher, M‐270, 100 µl) were added to each sample to pull down the biotin labeled Trp‐complex. After washing in washing buffer (10 mM Tris, 2 mM EDTA, 400 mM NaCl and 0.02% tween‐20), the immunocomplex was eluted by elution buffer ((biotin, 4 mg ml^−1^) in 25 mM Tris‐HCl containing 0.3 M NaCl (pH 8.5, as the elution buffer)) at room temperature for 5 min or by Qiazol lysis buffer for 10 min with frequent agitation. miR associated with the Trp complex was purified by miRNeasy column. RT‐qPCR was performed to quantify miR.

### Single‐Strand Gel Shift Assay

To identify the miRs that bind to Trp, miRs (Eurofins Genomics LLC, KY) was synthesized to perform the single‐strand shift assay, which was previously described.^[^ ^11^ ^]^ In brief, the miRs (10 µmol) were incubated with 200 µM of Trp at 37 °C for 30 min following electrophoresis on a 15% native polyacrylamide gel without SDS in 0.5x TBE buffer. The miRs were stained with ethidium bromide (0.5 mg ml^−1^) and visualized under ultraviolet (UV) light.

### Liposome Preparation

The neutral lipid DOPE (1,2‐dioleoyl‐sn‐glycero‐3‐phosphoethanolamine, Avanti, USA) and the cationic lipid DOTAP (N‐[1‐(2,3‐dioleoyloxy) propyl]‐N, N, N‐trimethylammonium chloride, Avanti, USA) were dissolved in chloroform to a working concentration of 10 mg ml^−1^. The lipid solutions were thoroughly mixed at a molar ratio of 1:1 and the solvent were evaporated under a nitrogen stream. The lipid film attached on the bottom of the tube was suspended in distilled water. Liposomes were prepared by bath sonication for 5 min until the lipid dispersed to clarity. An equal volume of 2xPBS was added and the mixture sonicated for 2 min. The mixture was sterilized by passing it through a 0.22 µm filter. Trp complex or miR were combined with the cationic lipid solution to the indicated concentration in a borosilicate glass tube (Fisher Scientific, USA). The mixture of lipid and Trp complex or miR was incubated for 5 min at room temperature and then the DOPE liposomes were stored until used in experiments.

### Cell Culture and Treatment

The mouse syngeneic colon tumor cell line (CT26) and human colonic epithelial SW620 cell line was maintained in Dulbecco's Modified Eagle Medium (DMEM, Gibco) media supplemented with 10% fetal bovine serum (FBS, Gibco) at 37 °C in a 5% CO_2_ environment.

Cells were Trp starved using Trp‐free medium for 12 h and then treated with PBS plus scrambled miR, Trp (200 µM) plus scrambled miR, miR‐193a‐3p (10 µM) or Trp complex (200 µM) for 24 h. Cells were harvested for further analysis.

### miR Extraction and RT‐qPCR

miR was extracted from cells and tissue using the miRNeasy Micro Kit (Qiagen) according to the manufacturer's instructions. Briefly, cells or tissue were dissolved in Qiazol lysis reagent. Samples were homogenized using a Fastprep homogenizer. The mixture was centrifuged at 12,000x g for 15 min after adding 140 µl chloroform. After transferring the upper layer to a fresh tube, a 1.5‐fold volume of 100% ethanol was added to the tube, mixed well, and transferred to a miRNeasy spin column. The flow‐through was discarded after centrifugation, and the column was rinsed using Buffer RWT and RPE sequentially. The total miR was eluted by RNase‐free water. The quality and quantity of the isolated miR was measured using a NanoDrop spectrophotometer.

For analysis of miR expression, 200 ng of total miR was reverse transcribed using a miScript II RT Kit (Qiagen). Q‐PCR was then performed on the BioRad CFX96 qPCR System and the comparative threshold cycle (Ct) value was recorded. Relative fold changes of gene expression were calculated based on the following formula: 2^−∆∆Ct^. Primers used include the following: mmu‐miR‐193a‐5p: TGGGTCTTTGCGGGCAAGATGA; mmu‐miR‐193a‐3p: AACTGGCCTACAAAGTCCCAGT; mmu‐miR‐107‐3p: ACTGCAGTGAGGGCACTTGTAG; mmu‐miR‐103‐3p: AGCAGCATTGTACAGGGCTATGA; Pre‐miR‐193a‐F: TGAGAGCTGGGTCTTTGCGG, Pre‐miR‐193a‐R: ACCGAGGACTGGGACTTTGTA; (snRNA)U6: GCTTCGGCAGCACATATACTAAAAT; anti‐miR‐103‐3p: UCGUCGUAACAUGUCCCGAUACU; anti‐miR‐107‐3p: UCGUCGUAACAUGUCCCGAUAGU; mcaprin1‐F: AAAGATGCCCTCGGCCAC; mcaprin1‐R: AGCGCTTCTGTTCTGCTTCT; hcaprin1‐F1: TGAAGCAGATTCTCGGGGTG; hcaprin1‐R1: TTCAGGTCAGTCCGCACTTC; hcaprin1‐F2: GAAGTGCGGACTGACCTGAA; hcaprin1‐R2: TCCTTCTGTGGTCGTTGCTC; hcaprin1‐F3: GAGCAACGACCACAGAAGGA, hcaprin1‐R3: GGGGAGCACTGAACTGAGAC; hGAPDH‐F: GTATGACAACAGCCTCAAGAT, hGAPDH‐R: GTCCTTCCACGATACCAAAG

### MicroRNA Chip Assay

Eight‐ to twelve‐week‐old male C57BL/6 mice mice (n = 5 per group) were provided the Trp‐free diet or pair diet (Trp, 0.3% w/w) for 3 days. The mice were sacrificed, and the liver, small intestine and large intestine collected. miR was extracted from all the tissues using the miRNeasy Micro Kit (Qiagen). The quantity and quality of the isolated miR was determined using the NanoDrop spectrophotometer. The miR was labeled using the Applied Biosystem FlashTag Biotin HSR RNA Labeling Kit (Thermo Fisher Scientific, Waltham, MA) followed by hybridization using the Applied Biosystem GeneChip miR 4.0 Array (Thermo Fisher Scientific, Waltham, MA) at 48 °C for 18 h. The array is designed with a 100% coverage of miRbase v20 and interrogates 30424 mature miRs as well as probe sets unique to mouse pre‐miR hairpin sequences. The arrays were processed following the manufacturer's recommended wash and stain protocol on an Affymetrix FS‐450 fluidics station and scanned on an Affymetrix GeneChip 7G scanner using Command Console 4.0. The resulting “.cel” files were imported into Transcription Analysis Software TAC (ThermoFisher, Waltham, MA) for analysis.

### KEGG Pathway and Gene Ontology Analysis of miRs

The Kyoto Encyclopedia of Genes and Genomes (KEGG) pathway annotation and Gene Ontology (GO) analysis was performed for three biological domains: biological process, molecular function, and cellular component. To identify KEGG pathways and GO enriched gene sets d that were potentially differentially expressed, the DIANA‐miRPath v.3 was used on 40 miRs (except mmu‐miR125b‐1 from the original 40 miRs). Interactions between the miRs and gene sets were identified in 3′UTR and CDS regions using the DIANA‐microT‐CDS with a default setting of P‐value threshold = 0.05 and MicroT threshold = 0.8.^[^ ^37^ ^]^

### Cloning and Constructs

The luciferase reporter containing 3243 bp of the caprin1 3′ UTR in the pEZX‐MT01 vector was purchased from GeneCopoeia (Cat#: MmiT072744‐MT06, MD, USA). A 318 bp region of the murine 3′UTR of Ptgs2 containing the putative miR‐103‐3p/miR‐107‐3p binding site was amplified by PCR using the following two primers: ACTAAGCGATCGCGAATTCCTCCCTCCGGTGTTTGTCCTT and GCTGGGTTACTAGTCTCGAGACTGAACTTGGACCCCTTTGTT. The PCR products were cloned into a luciferase reporter, pEZX‐MT01 (GeneCopoeia, Cat#: MmiT072744‐MT01, MD, USA) using the NEBuilder HiFi DNA Assembly Cloning Kit (NEB Biolabs, MA, USA). Constructs containing flag tag WT‐hAgo2 or mutant hAgo2 (pcDNA5 Flag‐hAgo2 P590G, pcDNA5 Flag‐hAgo2 K660S; R688S) were kindly provided as a gift from Dr. Ian J. MacRae (The Scripps Research Institute, CA, USA).^[^ ^7c^ ^]^

### Secrete‐Pair *Gaussia* Luciferase Assay Kit

A Gaussia luminescence assay was performed according to the instructions of the Secrete‐Pair *Gaussia* Luciferase Assay Kit. CT26 cells were transfected with 3′UTR caprin1 Gaussia luciferase reporter constructs^[^ ^12^ ^]^ or 3′UTR Ptgs2 Gaussia luciferase reporter constructs (0.5 µg) using lipofectamine 3000. After 24 h, the medium was changed, and the cells were transfected with or without Trp‐miR complex for another 24 h. The cell culture medium was collected and mixed with 1xBuffer GL‐S working solution at room temperature. The sample was evaluated for lucifer‐fluorescence using a microplate reader (Synergy HT, BioTek, Winooski, VT).

### Western Blotting

Cells or murine tissue were suspended in lysis buffer (20 mM Tris‐HCl, 150 mM NaCl, 1 mM EDTA, 1 mM EGTA and 1% Triton‐100) with proteinase inhibitor (cOmplete). Samples were homogenized using a needle and syringe or tissue grinder. Debris were discarded after centrifugation at 13,000x g for 10 min. Supernatants were collected and mixed with SDS loading buffer. After boiling for 5 min, 20–30 mg of each protein sample was loaded on an 8–12% SDS‐PAGE gel, electrophoresed and then transferred to a PVDF membrane (BioRad Laboratories, Inc., Hercules, CA). The membrane was incubated with primary antibody (Anti‐N‐cadherin, BD, 51–9001943; Anti‐β‐catenin, Cell Signaling, 9562L; Anti‐SNAIL, Abcam, ab63371; Anti‐β‐actin, Santa Cruz, sc‐47778; Anti‐c‐Myc, Santa Cruz, sc‐41; Anti‐cyclin D2, BD Biosciences, 554 201; Anti‐MVP, Proteintech, 16478‐1‐AP; Anti‐caprin1, Proteintech, 15112‐1‐AP; Anti‐COX2, Proteintech, 66351‐1‐Ig), overnight at 4 °C, rinsed and then incubated with Alexa Fluor‐790 (Invitrogen, A11357 and A11369) or iFluor800‐Streptavidin (AAT Bioquest, 16 976) conjugated secondary antibody for 1 h at room temperature. Bands were scanned and analyzed using an Odyssey imager (LiCor Inc, Lincoln, NE).

### Confocal Image Assay

CT26 cells were seeded and grown in fourwell chamber slides (Lab TekII, 154 526). After treatment of biotin labeled Trp (200 µM) or transfection of cy5‐miR (10 µM), the cells were washed with PBS and fixed using 4% paraformaldehyde for 20 min at room temperature. Next, the cells were blocked using 5% BSA solution containing 0.3% Triton X‐100, stained with an indicated primary antibody (Anti‐MVP, 1:400 dilution, Proteintech, or anti‐L‐Trp, 1:100 dilution, Immusmol, IS011) at 4 °C overnight. After rinsing twice in PBS, the specimens were stained with a fluorochrome‐conjugated secondary antibody (Invitrogen, A32723 and A32740) for 1 h in the dark. DAPI staining was conducted before the slides were covered with an anti‐fade mounting buffer. The images were visualized using a Nikon A1R‐A1 confocal microscope equipped with a digital image analysis system (Pixera, San Diego, CA).

### Isolation of CT26 Cell Derived Primary and Metastatic Cells

GFP‐CT26 cells were isolated from spleen and liver tissue of tumor bearing mice. Murine spleen and liver were resected, mixed with digestion buffer (HBSS containing 0.05% collagenase D (Worthington), 0.1% proteinase inhibitor cocktail (cOmplete), DNase I (2 mg ml^−1^), and 0.5% dispase (Gibco)), and minced using a dounce homogenizer. Cell suspensions were transferred to a fresh tube and rocked at 37 °C for 30 min. The digested samples were passed through a 100 µm strainer and the cells were pelleted by centrifugation at 300x g for 7 min. Erythrocytes were lysed using ACK buffer (0.15 M Ammonium chloride, 0.01 M Potassium bicarbonate and 0.1 mM EDTA) for 5 min. After a PBS wash, FACS sorting based on GFP positive cells was conducted using a flow cytometer (BD Biosciences, FACSCanto).

### Cell Viability

Cell viability was assessed using the Cell‐Quant AlamarBlue Cell Viability Reagent according to the manufacturer's instructions. In brief, 100 µl of cell suspension from each well were mixed with 10 µl of AlamarBlue Reagent for 2 h in a 5% CO_2_ incubator at 37 °C. The fluorescence of each sample was measured at an excitation wavelength at 530–560 nm and emission wavelength at 590 nm using a microplate reader (Synergy HT, BioTek, Winooski, VT).

### COX2 Overexpression

Crispr/Cas9 COX2 overexpression lentivirus (Santa cruz, sc‐422489) and packaging plasmid (pCMV delta R8.2 and pCMV‐Vddgeneddgene) were co‐transfected into HEK293T cells. After 48 h and 72 h, supernatants were collected and centrifuged to remove cell debris. Lentivirus particles were concentrated by mixing with PEG8000 overnight and resuspended in DPBS buffer. CT26 cells were infected with lentivirus for 6 h and then puromycin selection (2 mg mL^−1^) was performed 48 h post transduction. Cells were maintained for 7–14 days with antibiotics and a lentivirus producing monoclonal population was selected. HPCA or Rab11a knock out efficiency was determined by western blot.

### Circular Dichroism (CD) Measurements

CD experiments were carried out on a CD spectrometer (JASCO, USA) using a 10 mm path length cell. CD spectra of miRs (100 nM for miR‐193a‐3p) in the absence and presence of Trp (200 µM) were measured in 20 mM HEPES pH 7.2, 190 mM NaCl, 1 mM EDTA, 37.5 µg ml^−1^ BSA, 0.02% Tween‐20.

### Surface Plasmon Resonance Analysis

Interaction and binding analyses of Trp and miR‐193a‐3p were performed on an OpenSPR instrument (Nicoya Life Sciences) and all procedures were carried out according to the manufacturer's instructions. In brief, after cleaning a sensor chip with octyl b‐D‐glucopyranoside (40 mM) and CHAPS (20 mM), the chip was coated with streptavidin protein (200 µM, NTA). Biotin labeled miR‐193a‐3p (100 µM) was loaded onto the chip in binding buffer (10 mM HEPES, 190 mM NaCl, 1 mM EDTA and 0.02% Tween‐20). The amount of Trp (2, 20 and 200 µM) binding was measured by comparing the signal before and after ligand injection.

### Isothermal Titration Calorimetry Measurement

MiR‐193a‐3p (20 µM) was diluted in binding buffer to a final buffer composition of 20 mM HEPES pH 7.2, 190 mM NaCl, 1 mM EDTA, 37.5 µg ml^−1^ BSA, 0.02% Tween‐20. Trp was diluted in the same buffer to a final concentration of 200 µM. ITC measurements were collected using a MicroCal PEAQ‐ITC (Malvern Panalytical) at the indicated temperature. 200 µM ligand was titrated into binding buffer or 20 µM miR. One 0.4 µl injection was followed by 2 µl injections, with 150 seconds between injections and a 750‐rpm stirring speed. Dilution heats were subtracted from the raw titration data prior to data analysis. The data were evaluated using the MicroCal PEAQ‐ITC Evaluation software (Malvern).

### Trp Concentration Measurement

Tissue was homogenized in ice‐cold buffer of 50% methanol/50% PIPES‐EDTA (3 mM PIPES, 3 mM EDTA, pH 7.4) to quench the metabolites. Afterward, 200 µl ice‐cold chloroform was added and vortexed vigorously for 45 min at −20 °C. The upper layer was collected after the mixture was centrifuged at 21,000x g for 10 min at 4 °C. The bottom phase was re‐extracted by adding 200 µl methanol/PIPES buffer. The two obtained upper layers were combined and filtered using a 0.22 µm filter. High‐performance liquid chromatography (HPLC) was performed on an Agilent 1260 system equipped with a C18 column (4.6×150 mm, 5 µm, SB‐C18, ZORBAX). The mobile phase contained 15 mM potassium phosphate with 2.7% (v/v) acetonitrile, pH 6.4 and Trp was detected using an UV diode array detector (DAD) at a wavelength of 286 nm.

### Histological Analysis

Fresh liver tissues were resected and fixed in 10% formalin solution (SF93–20; Fisher Scientific, Fair Lawn, NJ) for 24 h at 4 °C. The specimens were then dehydrated by successive ethanol gradients of 70%, 80%, 95% and 100% for 45 min each, after which the tissues were immersed in xylene (Fisher) and embedded in paraffin. A microtome was used to cut ultrathin tissue slices (5 mm). Deparaffinization and rehydration were performed using xylene and ethanol gradients starting at 100% followed by 95%, 80% and 70% concentrations. Sections were stained with hematoxylin and eosin and scanned using the Aperio ScanScope slide scanner.

### In Vitro Ago2 Activity Assay

An Ago2 in vitro cleavage assay was performed according to a previous publication with minor modifications.^[^ ^21^, ^38^ ^]^ Murine Ago2 recombinant protein was purchased from SinoBiological Inc. (Cat#: 50683‐M07B) and human Ago2 recombinant protein was provided as a gift from Dr. Ian J. MacRae (The Scripps Research Institute, CA, USA).^[^ ^7c^ ^]^ In brief, Ago2 protein (1 µM) was incubated with 100 nM miR‐193a‐3p or Trp (200 µM)‐miR‐193a‐3p (500 nM) complex for RISC assembly in reaction buffer (25 mM HEPESKOH, pH 7.5, 5 mM MgCl_2_, 50 mM KCl, 5 mM DTT, 0.2 mM EDTA, 0.05 mg ml^−1^ BSA and 5 u µl^−1^ RNase inhibitor resin) at 37 °C for 2 h and followed by adding 2.5 µg RNA derived from SW620 or CT26 cell lysates. After 1 h in a 37 °C water bath, caprin1 cleavage product was analyzed by RT‐qPCR using the mixture as template.

### Fluorescence In Situ Hybridization

Colon tissue slides from colon cancer metastatic liver patients were purchased from AMSBIO (Cambridge, Massachusetts). After deparaffinization, sections were prehybridized for 2 h at 50 °C in prehybridization buffer (9 mM NaCl, 5 mM EDTA and 20 mM Tri‐HCl, pH 8.0). Anti‐miR‐193a‐3p probes were biotin‐labeled using a PHOTOPROBE Biotin for Nucleic Acid Labeling kit (Cat# SP‐1000, VECTOR, Newark, California). The section was then hybridized with probes (2 µg) in hybridization buffer overnight (18 h) at 50 °C. Sections were washed 2 times in SSC buffer (150 mM NaCl, 17 mM sodium citrate, pH 7.0) at 37 °C for 15 min, followed by one wash in SSC buffer at 50 °C for another 15 min and then finally washed four times with PBST (PBS containing 0.01% Tween‐20). Tissue sections were co‐incubated with anti‐L‐Trp antibody (1:100 dilution) for 1 h at room temperature and then stained with fluorochrome‐conjugated streptavidin (Streptavidin Alexa Fluor 488, Thermo Fisher) and goat anti‐rabbit antibody (1:600 dilution). DAPI staining was conducted before the slides had coverslips placed on them with anti‐fade mounting buffer. The images were visualized using a Nikon A1R‐A1 confocal microscope equipped with a digital image analysis system.

### Clinical Samples

All clinical samples, including liver and colon tissue, were collected in the Department of Surgery, Huai'an First People's Hospital, Huai'an, Jiangsu, China with written informed consent from all human participants. Approval for the study was granted by the Institute Research Ethics Committee at the Health Department of Huai'an. Stages of colon cancer reflect the extent of cancer involvement according to the criteria from the National Cancer Institute (http://www.cancer.gov/types/colorectal/patient/colon‐treatment‐pdq#section/_112).

### Statistical Analysis

To determine significant difference of the results, SPSS 16.0 software using one‐ or two‐ way ANOVA and Student *t*‐test (^*^ *p*<0.05, ^**^ *p*<0.01, ^***^ *p*<0.001 and ^****^ *p*<0.0001) was employed. A *p*‐value greater than 0.05 was marked as not statistically significant (NS). The reported “‘n”’ in animal and human studies represents the number of animals and human subjects. Data are representative of at least three independent experiments.

### Ethics Declarations

Animal care was performed according to the Institute for Laboratory Animal Research (ILAR) regulations, and all animal procedures (IACUC 21918) were approved by the University of Louisville Institutional Animal Care and Use Committee (Louisville, KY).

All clinical samples, including liver and colon tissue, were collected in the Department of Surgery, Huai'an First People's Hospital, Huai'an, Jiangsu, China with written informed consent from all human participants. Approval for the study (KY‐2023‐120‐01) was granted by the Institute Research Ethics Committee at the Health Department of Huai'an. Stages of colon cancer reflect the extent of cancer involvement according to the criteria from the National Cancer Institute (http://www.cancer.gov/types/colorectal/patient/colon‐treatment‐pdq#section/_112).

## Conflict of Interest

The authors declare no conflict of interest.

## Author Contributions

F.X., Y.R., and Y.T. contributed equally to this work. F.X. and H‐G.Z. designed the study, analyzed, and interpreted the data and prepared the manuscript; Y.T., Y.R., J.M., J.T., K.S., and L.Z. performed the experiments and interpreted the data; J.W.P. and J.H.H performed bioinformatic analysis and J. Y., and G.D. interpreted the findings.

## Supporting information

Supporting Information

## Data Availability

The data that support the findings of this study are available from the corresponding author upon reasonable request.
