# Supplementary material for: Tryptophan As a New Member of RNA‐Induced Silencing Complexes Prevents Colon Cancer Liver Metastasis
Source: Adv Sci (Weinh). 2024 Jun 20;11(31):2307937. doi: 10.1002/advs.202307937 (PMC11336974; doi:10.1002/advs.202307937)
Supplement: Supplementary file 1 — Supporting Information [file ADVS-11-2307937-s001.pdf]

ADVANCED SCIENCE
Open Access

Supporting Information

for *Adv. Sci.*, DOI 10.1002/advs.202307937

Tryptophan As a New Member of RNA-Induced Silencing Complexes Prevents Colon Cancer Liver Metastasis

Fangyi Xu, Yi Ren, Yun Teng, Jingyao Mu, Jie Tang, Kumaran Sundaram, Lifeng Zhang, Juw Won Park, Jae Yeon Hwang, Jun Yan, Gerald Dryden and Huang-Ge Zhang**

Supporting Information

Tryptophan as a new member of RNA-induced silencing complexes prevents colon cancer liver metastasis

Fangyi Xu[#], Yi Ren[#], Yun Teng[#], Jingyao Mu, Jie Tang, Kumaran Sundaram, Lifeng Zhang, Juw Won Park, Jae Yeon Hwang, Jun Yan, Gerald Dryden, and Huang-Ge Zhang**

Figure supplementary legends

Fig. S1 (related to Figure 1). A lower level of tryptophan alters the miR profile in the Trp/miR complex

(A). Biotin labeled Trp complexes from LI tissue of BALB/c mice given biotin labeled Trp (300 mg/kg) by gavage was pulled down using streptavidin coated beads. The isolated complexes were packed in DOPE liposomes and then CT26 cells were transfected with Trp complex (200 µM) for 24 h. Trp complexes treated with RNase A (2 µg/ml), DNase (1 µg/ml) and protease (50 µg/ml) were used as controls. Cell viabilities were assessed using Cell-Quant™ AlamarBlue Cell Viability Reagent. P values were calculated by means of an ANOVA test. *$P < 0.05$. Representative images were shown on the bottom.

(B). CT26 cells were cultured in Trp depleted or Trp supplemented (50 µM or 200 µM) medium for 48 h. Total RNA including miRs were isolated and the level of pri- or mature miR-193a-3p or miR-193a-5p were determined by RT-qPCR. P values were calculated by means of an ANOVA test. *$P < 0.05$, NS, no significance.

(C). Genotyping analysis of offspring from IDO1 wild-type (WT) and IDO1 knockout (KO) mice.

(D). Trp level was detected in the large intestine tissue derived from WT and IDO1-KO mouse. HPLC was performed to quantify Trp concentration.

(E). Expression of miR-193a-3p in the LI tissue of WT or IDO1 KO mice and LI tissue derived exosomes determined using RT-qPCR. P values were calculated by means of an ANOVA test. *$P < 0.05$, **$P < 0.01$.

(F). miR-193a-3p, miR-17-5p, miR-106a-5p, miR-378a-3p and miR-29a-3p were randomly selected from the data as presented in Fig. 1C. The RT-qPCR was done to confirm the results generated by the miR chip. Rnu6 was used as internal control.

(G-H). Trp (200 μM) was co-incubated with miR-222-3p, miR-468-5p, miR-151a-3p, miR-193a-3p and miR-31-5p, miR-15b-5p, miR-125a-5p and miR-92a-3p (10 μM) at 37°C for 15 min. Native PAGE was performed and the gel was stained with EB and the bands were visualized using UV light. A representative image (n=3) is shown.

(I). CD spectra were performed, and details can be found in the Methods section. CD signal of miR-193a-3p (100 nM) mixed with Phe (Phenylalanine, 200 μM), miR-193a-5p (100 nM) mixed with Trp or miR-193a-mutation (100 nM) mixed with Trp (200 μM) is shown in the figures.

(J). Proposed schematic model showing Trp binding with miR-193a-3p and the mutation strategy of miR-193a-3p at the positions indicated.

(K). ITC measurements for the binding of tryptophan (200 μM) to miR-193a-3p (10 μM). Details can be found in the Methods section.

(L). ITC measurements for the binding of Trp (200 μM) to mutant miR-193a-3p (10 μM).

Fig. S2 (related to Figure 2). miRs in the Trp/miR complex work as a group and have a stronger activity on the regulation of miR targeted gene expression than individual miRs

(A). CT26 cells were Trp starved for 12 h and then treated with equal amounts of free-form biotin or biotin labeled Trp for 12 h (200 μM). Trp complexes were pulled down with streptavidin beads and the miRNA interacting with Trp was isolated. miR levels from CT26 cells pulled down by biotin-Trp were analyzed using a miR chip. Pathway enrichment analysis of miR associated with the Trp complex based on the results of the miR chip array. The top 30 enriched pathway terms are displayed. The P-value indicates the enrichment level of the pathway term and ranges from 0 to 1. A lesser P-value indicates greater intensiveness. The size of the ball represents the gene numbers related to the pathway regulated by miR.

(B). CT26 cells were transfected with Trp complex (200 µM) pulled down from mouse LI tissue for 24 h. Scrambled miR (10 µM, the first row shown), Trp complexes treated with RNase A (2 µg/ml, the second row shown) and anti-sense miR-193a-3p (10 µM, the fourth row shown) were used as controls. Western blots were performed, and band intensity quantified and normalized to β-actin. The results are presented between the panels (n=3).

(C). BALB/c mice (n=5 per group) were treated with a Trp-free diet for 3 days and then mice were gavage given biotin labeled Trp (300 mg/kg). After 24 h, mice were sacrificed, and the Trp complex from liver (left panel), large intestine tissue (LI, middle panel) and small intestine (SI, right panel) tissue were pulled down using streptavidin beads. After being packed into DOPE liposomes (DOTAP: DOPE=1:1 in mol ratio), the complexes were used to transfect CT26 cells for 24 h. Complexes treated with RNase A (10 µg/ml) and anti-sense miR-193a-3p (10 µM) were used as controls. Cell viabilities were assessed using Cell-Quant™ AlamarBlue Cell Viability Reagent. P values were calculated by means of an ANOVA test. **P < 0.01, ***P < 0.001.

(D). CT26 cells were transfected with scrambled miR, Trp plus scrambled RNA, Trp-miR-193a-3p or Trp complex (200 µM) at indicated times. Cell migration was assessed using a wound healing assay. Wound closure is expressed as the remaining area not covered by the cells. Representative bright-field images show that the Trp complex resulted in significantly decreased migration speed (Left). Migration rate was evaluated as the percentage wound area at 0 h and 24 h (Right). P values were calculated by means of an ANOVA test. *P < 0.05, **P < 0.01, ***P < 0.001.

(E). CT26 cells were transfected with scrambled miR, Trp plus scrambled RNA, Trp-miR-193a-3p or Trp complex (200 µM) for 36 h. Cell invasion was assessed using the transwell assay system. Representative bright-field images are shown, and cell invasion ability was calculated by counting cells per field. P values were calculated by means of an ANOVA test. *** P < 0.001 and ****P < 0.0001.

(F). Schematic diagram of the putative binding sites of miR-193a-3p, miR-103-3p/107-3p in caprin1-3′-UTR and of miR-103/107-3p in Ptgs2 (COX2) 3′-UTR.

(G). CT26 cells were transfected with Trp complex or Trp plus triple miRs (miR-193a-3p/103-3p/107-3p) for 24h. Scrambled miR and Trp plus scrambled RNA was used as controls. RT-qPCR

were performed to quantify the level of caprin-1 and cox2. Data represents the mean ± SEM. P values were calculated by means of the Student's t test. *P < 0.05, **P < 0.01, ***P < 0.001, ****P < 0.0001.

(H). CT26 cells were transfected with Trp complex or Trp plus triple miRs (miR-193a-3p/103-3p/107-3p) for 24h. Scrambled miR and Trp plus scrambled RNA was used as controls. Western blots were performed, band intensity quantified and normalized to β-actin. The results are presented between the panels (n=3).

(I-J). CT26 cells were transfected with scrambled miR (10 μM), miR-193a-3p (10 μM), Trp complex (200 μM), Trp complex plus antisense-miR193a, antisense-miR-103-3p (10 μM) or antisense-miR-107-3p (10 μM) for 24 h. Cell medium was collected and cytokine arrays were performed. The heat map indicates the level of all the cytokines in cell medium of each group. The red color indicates the cytokine is upregulated while the green color indicates the cytokine is downregulated. The cytokine levels of IL-1β, IL-4, IL-6, IL-10 and IL-12 in the cell medium were quantitatively analyzed with an ELISA. Data represent the mean ± SEM. P values were calculated by means of the Student's t test. *P < 0.05.

Fig. S3 (related to Figure 3). Trp promotes miR-193a-mediated targeted mRNA cleavage by enhancing Ago2 RNase activity

(A). CT26 cells were transfected with Trp plus miR-193a-3p after the cells were pre-treated with or without BCI-137 (30 μM) for 24h. Scrambled miR and Trp plus scrambled RNA was used as controls. Cell viabilities were assessed using Cell-Quant™ AlamarBlue Cell Viability Reagent. P values were calculated by means of an ANOVA test. *P < 0.05.

(B). An in vitro Ago2 activity assay was performed using Ago2 and miR-193a-3p in the presence of Trp, followed by adding mRNA from SW620 cells. Expression of caprin1 was determined by RT-qPCR. Specific PCR primers that can amplify the indicated caprin1 nucleotides sequences at position 161 to 487 bp, 487 to 1366 bp and 1366 to 1959 bp were used and are shown on the schematic plot. P values were calculated by means of an ANOVA test. *P < 0.05, ***P < 0.001, NS, no significance.

(C). An in vitro Ago2 activity assay was performed using Ago2 and guide miR-193a-3p in the presence of Trp at the indicated concentration, followed by adding mRNA from CT26 cells. Expression of caprin1 was determined by RT-qPCR. P values were calculated by means of an ANOVA test. $*P < 0.05$ and $**P < 0.01$.

(D). CT26 cells were transfected with miR-193a-3p in the presence of Trp at different concentrations. Total RNA was isolated from the cells and caprin1 expression was detected by RT-qPCR. P values were calculated by means of an ANOVA test. $*P < 0.05$.

(E). CT26 cells were transfected with Flag-Ago2-WT or Flag-Ago2 Mutant (P590G and R688S) plasmid for 48 hours. Transfected CT26 cells were cultured in Trp depleted medium for 12h, and then treated with PBS or Trp (200 μM) for culturing additional 12 h before the cells were harvested. Ago2 complex was pulled down by protein G beads cross-linked with anti-Flag M2 antibody (Sigma) and eluted with 3xFlag peptide (Rockland Immunochemicals). An Ago2 activity assay was performed using Ago2 complex in the presence of guide miR-193a-3p, followed by adding mRNA from CT26 cells. Expression of caprin1 was determined by RT-qPCR. P values were calculated by means of an ANOVA test. $*P < 0.05$, NS, no significance.

(F). Trp concentration in the serum of patients in cancer stage I/II (circles) and stage III/IV (squares) were determined by HPLC. NS, no significance.

(G). Fluorescence in situ hybridization of tumor sections from CT26 cell induced mice metastatic liver samples stained with Ago2 (green) and overlayed with Trp (red) and DAPI (blue). Five random fields were photographed, and representative images are shown. Scale bars, 40 μm. Percentage of co-localization intensity was calculated. P values were calculated by means of an ANOVA test. $**P < 0.01$.

(H). Fluorescent images of tumor sections from CT26 cell induced mice metastatic liver samples stained with Ago2 (green) and overlayed with Trp (red) and DAPI (blue). Five random fields were photographed, and representative images are shown. Scale bars, 40 μm. Percentage of co-localization intensity was calculated. P values were calculated by means of an ANOVA test. $*P < 0.05$

(I). Fluorescence images of tumor sections from CT26 cell induced mice metastatic liver samples stained with COX2 (green) and overlayed with caprin1 (red) and DAPI (blue). Five random fields were photographed, and representative images are shown. Scale bars, 20 μm. Fluorescent intensity of caprin1 or COX2 was calculated. P values were calculated by means of an ANOVA test. **P < 0.01, ***P < 0.001.

(J-K). Gene expression levels of caprin1 and Ptgs2 (COX2) in colon primary tumors compared to normal tissues (TCGA-COAD). P values were calculated by means of an ANOVA test. ***P < 0.001.

(L-M). Gene expression levels of miR-193a (TCGA-COAD) and miR-107 (dataset: GSE73487) in colon primary tumors compared to normal tissues. P values were calculated by means of an ANOVA test. *P < 0.05, ***P < 0.001.

Fig. S4 (related to Fig. 4). Trp organized miR-193a-3p/miR-103-3p/miR-107-3p works as a group to inhibit colon cancer metastasis to the liver

(A). BALB/c mice were given an intrasplenic injection of CT26 cells. Liver CT26 cells were sorted based on the GFP positive population and CT26 cell derived exosomes were pulled down using streptavidin beads. miR-193a-3p expression in tumor cells was quantified by RT-qPCR. The value is normalized to the miRNA level on Day 0. P values were calculated by means of an ANOVA test. *P < 0.05, ****P < 0.0001.

(B). Trp levels detected in the liver tissue derived from BALB/c mice given an intrasplenic injection of CT26 cells and a simultaneous *i.v.* administration of liposome containing scrambled miR, Trp plus scrambled miR, miR-193a-3p or Trp complex. HPLC was performed to quantify Trp concentration. P values were calculated by means of an ANOVA test. *P < 0.05, ***P < 0.001.

(C-D). Splenic injection of CT26 cells transfected using Crispr/Cas9 COX2 overexpression lentivirus particles was performed using BALB/c mice. Representative livers (metastatic nodules indicated with blue arrows) from tumor-bearing mice (n=5) *i.v.* injected with Trp complex treated with RNase, Trp complex, and Trp complex plus antisense-miR-193a-3p, antisense-miR-193a-3p/107-3p/103-3p, antisense-miR-107-3p/miR-103-3p. Arrows indicate metastasis tumor foci.

(E-F). Liver weight (right, top panel) and number of metastatic foci in liver were quantitatively analyzed. P values were calculated by means of an ANOVA test. *$P < 0.05$, ***$P < 0.001$.

(G). H&E-stained sections of tumor-bearing livers (scale bar 100 µm) from mice treated with Trp complex treated with RNase, Trp complex and Trp complex plus antisense-miR-193a-3p, antisense-miR-193a-3p/107-3p/103-3p, antisense-miR-107-3p/miR-103-3p. Arrows indicate metastasis tumor foci.

(H). Western blot analysis showing the level of COX2 in liver cancer tissue from mice *i.v.* injected with Trp complex (200 µM) pretreated with RNase, Trp complex, and Trp complex plus antisense-miR-193a-3p, antisense-miR-193a-3p/107-3p/103-3p, antisense-miR-107-3p or antisense-miR-103-3p (10 µM). Band intensities are normalized to β-actin and the results are presented between the panels.

Fig. S1

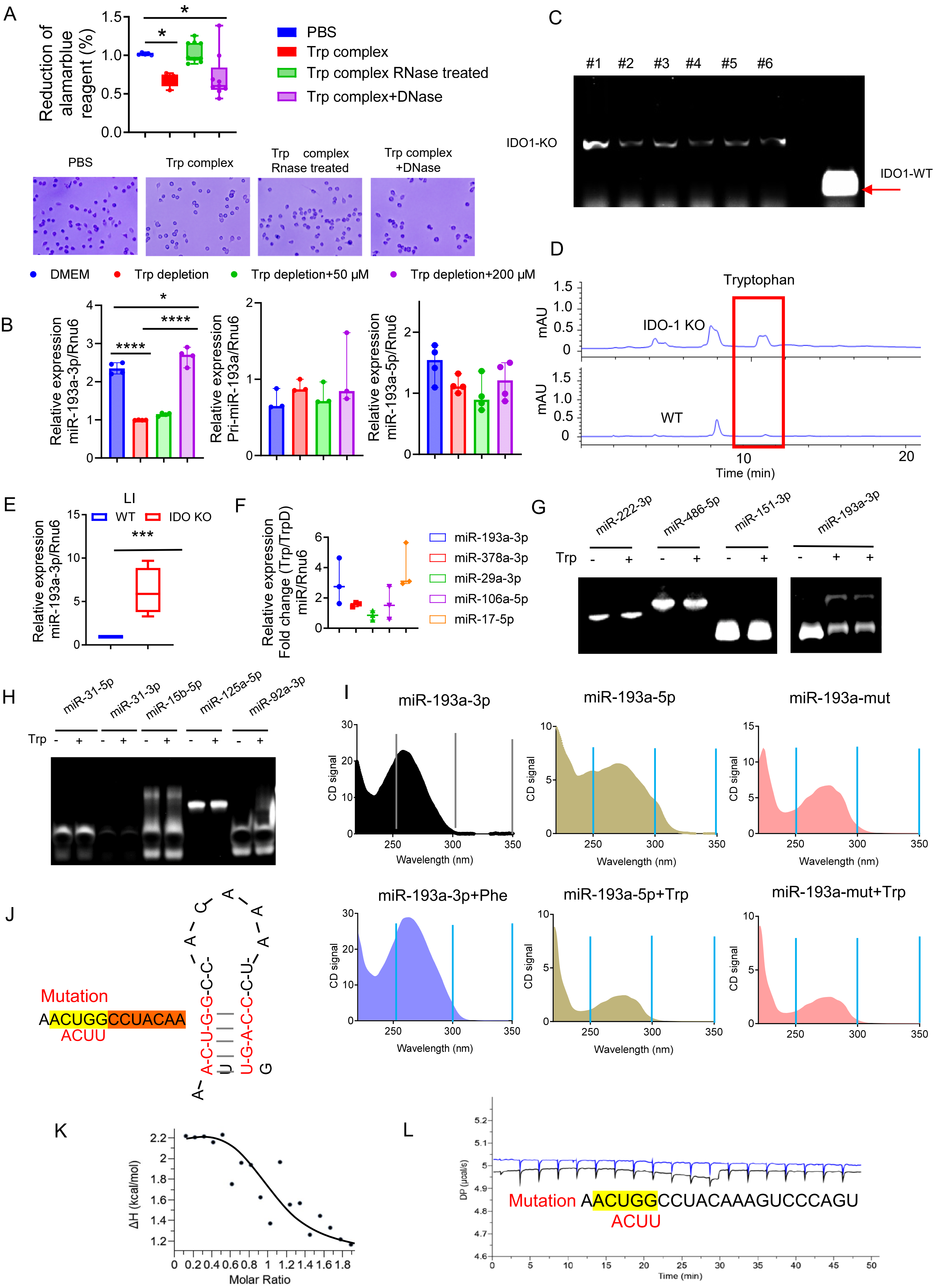
A
Reduction of alamarblue reagent (%)
PBS
Trp complex
Trp complex RNase treated
Trp complex+DNase
Trp complex Rnase treated
Trp complex +DNase
C
#1 #2 #3 #4 #5 #6
IDO1-KO
IDO1-WT
D
Tryptophan
IDO-1 KO
WT
mAU
Time (min)
DMEM
Trp depletion
Trp depletion+50 μM
Trp depletion+200 μM
B
Relative expression miR-193a-3p/Rnu6
Relative expression Pri-miR-193a/Rnu6
Relative expression miR-193a-5p/Rnu6
E
LI
WT
IDO KO
Relative expression miR-193a-3p/Rnu6
F
Relative expression Fold change (Trp/TrpD) miR/Rnu6
miR-193a-3p
miR-378a-3p
miR-29a-3p
miR-106a-5p
miR-17-5p
G
miR-222-3p
miR-486-5p
miR-151-3p
miR-193a-3p
Trp
H
miR-31-5p
miR-31-3p
miR-15b-5p
miR-125a-5p
miR-92a-3p
Trp
I
miR-193a-3p
miR-193a-5p
miR-193a-mut
miR-193a-3p+Phe
miR-193a-5p+Trp
miR-193a-mut+Trp
CD signal
Wavelength (nm)
J
Mutation
AACUGGCCUACAA
ACUU
K
ΔH (kcal/mol)
Molar Ratio
L
DP (μcal/s)
Time (min)
Mutation AACUGGCCUACAAAGUCCCAGU
ACUU

Fig. S2

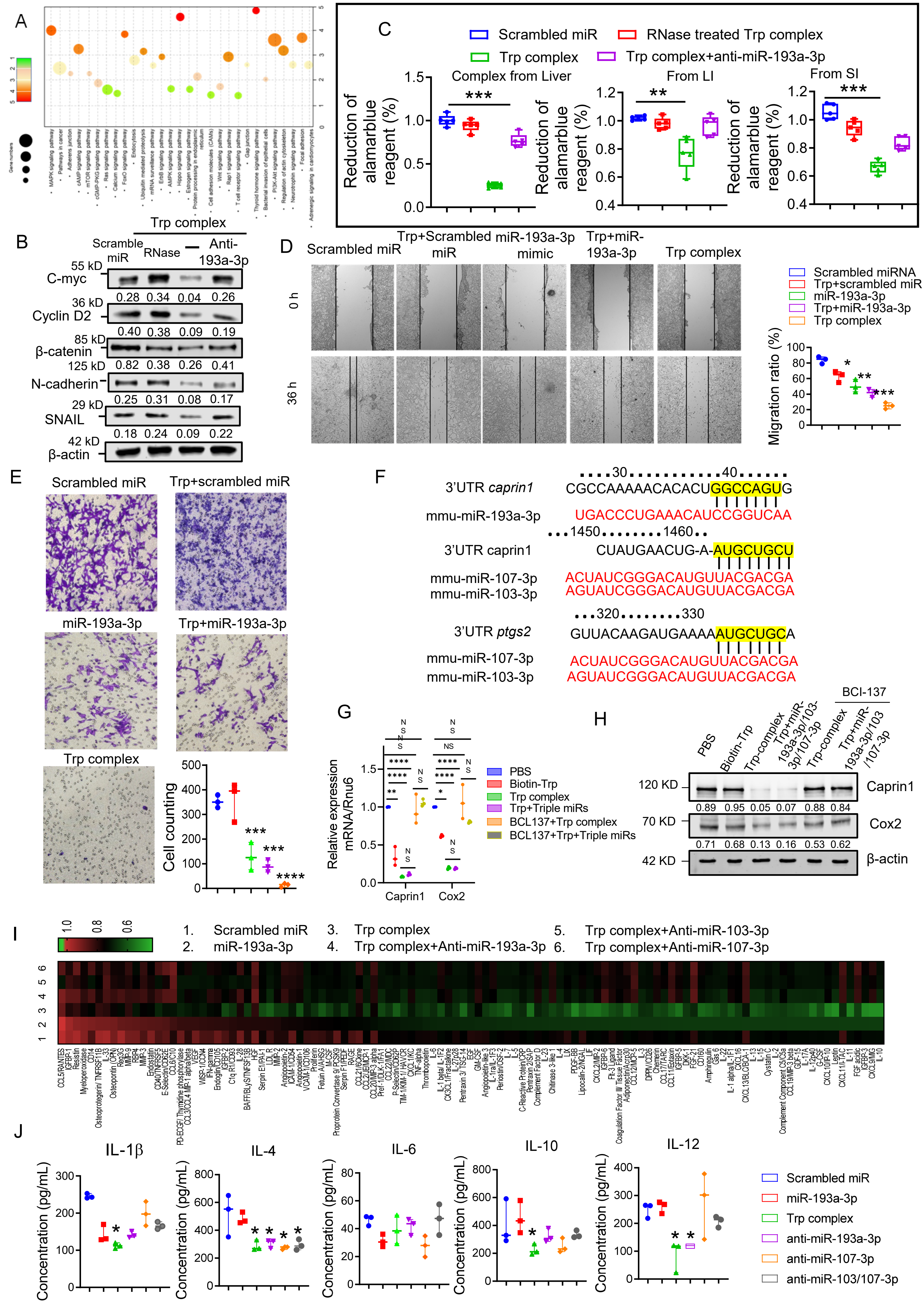

A
B
Trp complex
Scramble miR
RNase
Anti-193a-3p
55 kD
C-myc
0.28 0.34 0.04 0.26
36 kD
Cyclin D2
0.40 0.38 0.09 0.19
85 kD
β-catenin
0.82 0.38 0.26 0.41
125 kD
N-cadherin
0.25 0.31 0.08 0.17
29 kD
SNAIL
0.18 0.24 0.09 0.22
42 kD
β-actin
C
Scrambled miR
RNase treated Trp complex
Trp complex
Trp complex+anti-miR-193a-3p
Complex from Liver
From LI
From SI
Reduction of alamarblue reagent (%)
D
Scrambled miR
Trp+Scrambled miR
miR-193a-3p mimic
Trp+miR-193a-3p
Trp complex
0 h
36 h
Scrambled miRNA
Trp+scrambled miR
miR-193a-3p
Trp+miR-193a-3p
Trp complex
Migration ratio (%)
E
Scrambled miR
Trp+scrambled miR
miR-193a-3p
Trp+miR-193a-3p
Trp complex
Cell counting
F
3'UTR caprin1
CGCCAAAAACACACUGGCCAGUG
mmu-miR-193a-3p
UGACCCUGAAACAUCCGGUCAA
3'UTR caprin1
CUAUGAACUG-A-AUGCUGCU
mmu-miR-107-3p
ACUAUCGGGACAUGUUACGACGA
mmu-miR-103-3p
AGUAUCGGGACAUGUUACGACGA
3'UTR ptgs2
GUUACAAGAUGAAAAAUGCUGCA
mmu-miR-107-3p
ACUAUCGGGACAUGUUACGACGA
mmu-miR-103-3p
AGUAUCGGGACAUGUUACGACGA
G
Relative expression mRNA/Rnu6
Caprin1
Cox2
PBS
Biotin-Trp
Trp complex
Trp+Triple miRs
BCL137+Trp complex
BCL137+Trp+Triple miRs
H
BCI-137
PBS
Biotin-Trp
Trp-complex
Trp+miR-193a-3p/103-3p/107-3p
Trp-complex
Trp+miR-193a-3p/103/107-3p
120 KD
Caprin1
0.89 0.95 0.05 0.07 0.88 0.84
70 KD
Cox2
0.71 0.68 0.13 0.16 0.53 0.62
42 KD
β-actin
I
1. Scrambled miR
2. miR-193a-3p
3. Trp complex
4. Trp complex+Anti-miR-193a-3p
5. Trp complex+Anti-miR-103-3p
6. Trp complex+Anti-miR-107-3p
J
IL-1β
IL-4
IL-6
IL-10
IL-12
Concentration (pg/mL)
Scrambled miR
miR-193a-3p
Trp complex
anti-miR-193a-3p
anti-miR-107-3p
anti-miR-103/107-3p

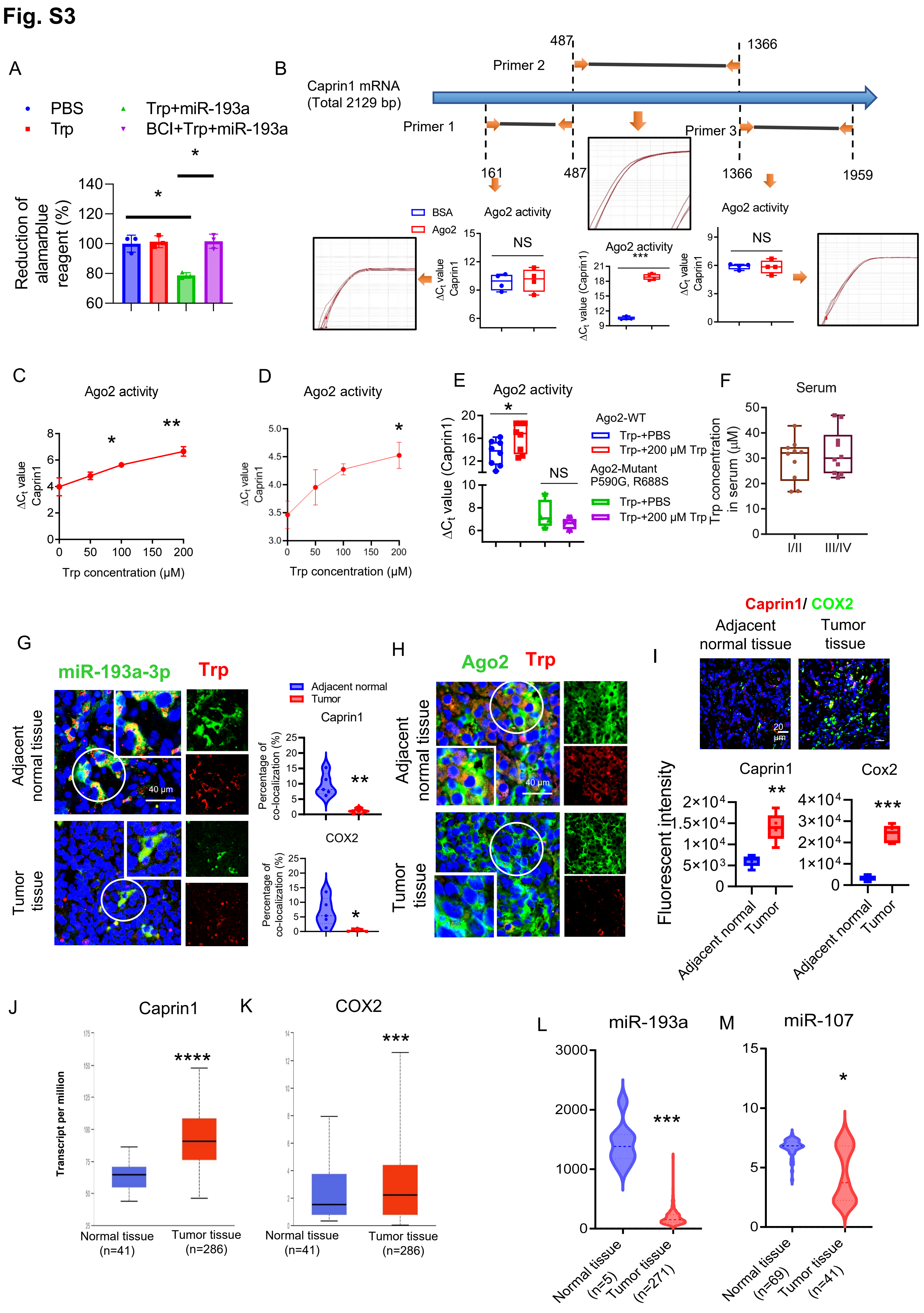
Fig. S3
A
PBS
Trp
Trp+miR-193a
BCI+Trp+miR-193a
Reduction of alamarblue reagent (%)
B
Caprin1 mRNA
(Total 2129 bp)
Primer 1
Primer 2
Primer 3
161
487
1366
1959
BSA
Ago2
Ago2 activity
NS
***
ΔCt value Caprin1
ΔCt value (Caprin1)
C
Ago2 activity
ΔCt value Caprin1
Trp concentration (μM)
D
Ago2 activity
Trp concentration (μM)
E
Ago2 activity
ΔCt value (Caprin1)
Ago2-WT
Trp-+PBS
Trp-+200 μM Trp
Ago2-Mutant
P590G, R688S
F
Serum
Trp concentration in serum (μM)
I/II
III/IV
G
miR-193a-3p
Trp
Adjacent normal tissue
Tumor tissue
40 μm
Adjacent normal
Tumor
Caprin1
COX2
Percentage of co-localization (%)
H
Ago2
Trp
I
Caprin1/ COX2
Adjacent normal tissue
Tumor tissue
Fluorescent intensity
Cox2
J
Caprin1
Transcript per million
Normal tissue (n=41)
Tumor tissue (n=286)
K
COX2
L
miR-193a
Normal tissue (n=5)
Tumor tissue (n=271)
M
miR-107
Normal tissue (n=69)
Tumor tissue (n=41)

Fig. S4

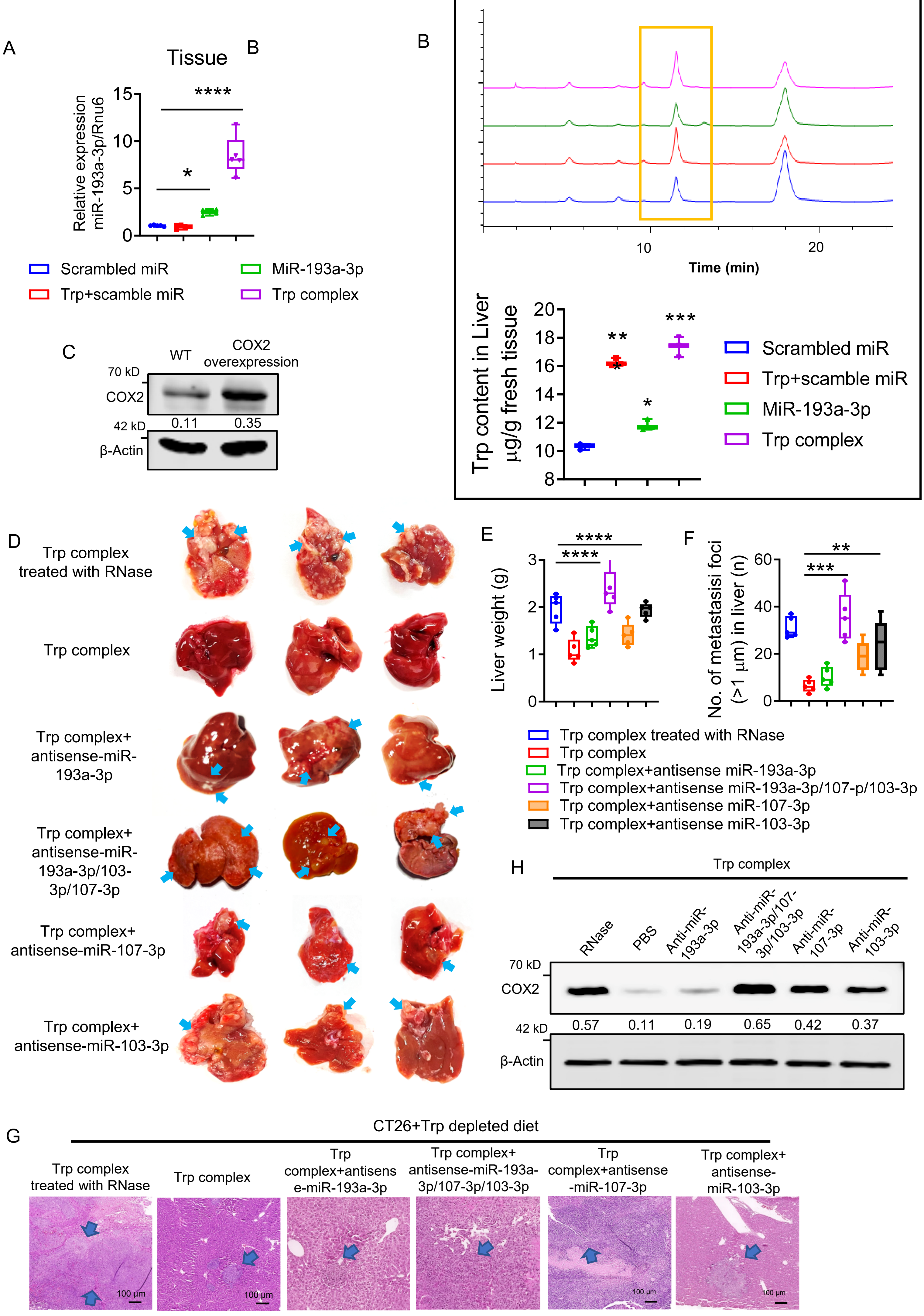
A
Tissue
B
Relative expression miR-193a-3p/Rnu6
****
*
Scrambled miR
Trp+scamble miR
MiR-193a-3p
Trp complex
B
Time (min)
10
20
Trp content in Liver μg/g fresh tissue
**
***
*
Scrambled miR
Trp+scamble miR
MiR-193a-3p
Trp complex
C
WT
COX2 overexpression
70 kD
COX2
42 kD
0.11
0.35
β-Actin
D
Trp complex treated with RNase
Trp complex
Trp complex+ antisense-miR-193a-3p
Trp complex+ antisense-miR-193a-3p/103-3p/107-3p
Trp complex+ antisense-miR-107-3p
Trp complex+ antisense-miR-103-3p
E
Liver weight (g)
****
****
F
No. of metastasisi foci (>1 μm) in liver (n)
**
***
Trp complex treated with RNase
Trp complex
Trp complex+antisense miR-193a-3p
Trp complex+antisense miR-193a-3p/107-p/103-3p
Trp complex+antisense miR-107-3p
Trp complex+antisense miR-103-3p
H
Trp complex
RNase
PBS
Anti-miR-193a-3p
Anti-miR-193a-3p/107-3p/103-3p
Anti-miR-107-3p
Anti-miR-103-3p
70 kD
COX2
42 kD
0.57
0.11
0.19
0.65
0.42
0.37
β-Actin
G
CT26+Trp depleted diet
Trp complex treated with RNase
Trp complex
Trp complex+antisense-miR-193a-3p
Trp complex+ antisense-miR-193a-3p/107-3p/103-3p
Trp complex+antisense-miR-107-3p
Trp complex+ antisense-miR-103-3p
100 μm

Table S1 (related to Figure 1) Clinical characteristics of 20 patients with colon cancer

Case#	Sex	Age	Stage	Pathology	pTNM: T	pTNM: N	pTNM: M	Grade
2022002733	Female	54	IIB	Adenocarcinoma	4	0	0	Moderate-differentiated
2022007602	Male	73	IIA	Adenocarcinoma	3	0	0	Moderate-differentiated
2022008749	Female	64	IIA	Adenocarcinoma	3	0	0	Moderate-differentiated
2022011641	Female	55	IIB	Adenocarcinoma	4	0	0	Moderate-differentiated
2022011769	Female	53	IIA	Adenocarcinoma	3	0	0	Moderate-differentiated
2022012068	Male	61	I	Adenocarcinoma	2	0	0	Moderate-differentiated
2022012664	Female	54	IIA	Adenocarcinoma	3	0	0	Moderate-differentiated
2022013221	Female	64	IIA	Adenocarcinoma	3	0	0	Moderate-differentiated
2022015878	Male	59	I	Adenocarcinoma	2	0	0	Moderate-differentiated
2022002594	Male	51	IIIB	Adenocarcinoma	4a	1a	0	Moderate-differentiated
2022003618	Male	54	IIIB	Mucinous adenocarcinoma	3	1a	0	Moderate-differentiated
2022006807	Male	53	IIIB	Adenocarcinoma	3	1b	0	low or moderate -differentiated
2022008054	Female	64	IVA	Adenocarcinoma	3	0	1	Moderate-differentiated
2022008447	Male	59	IIIB	Mucinous adenocarcinoma	3	1a	0	Moderate-differentiated
2022011541	Male	50	IIIB	Adenocarcinoma	3	2a	0	Moderate-differentiated
2022011981	Male	75	IIIB	Adenocarcinoma	3	1a	0	Moderate-differentiated
2022011983	Male	75	IIIB	Adenocarcinoma	3	2a	0	Moderate-differentiated
2022013628	Male	63	IIIC	Adenocarcinoma	4	2	0	low or moderate -differentiated
2022016533	Female	48	IIIB	Adenocarcinoma	3	2a	0	Moderate-differentiated
2022015396	Male	72	IIIB	Adenocarcinoma	3	1b	0	Moderate-differentiated

Table S2 (related to Figure 1) Clinical data of 20 patients with colon cancer

	Tryptophan			miR-193a-3p (ΔCt)		miR-107-3p(ΔCt)		miR-103-3p(ΔCt)	
Case#	Serum (μmol/L)	Tumor (μmol/g)	Adjacent Normal (μmol/g)	Tumor	Adjacent Normal	Tumor	Adjacent Normal	Tumor	Adjacent Normal
2022002733	42.8	0.0434	0.1193	10.7767	9.8733	11.8813	10.3670	11.3155	10.8854
2022007602	17.03	0.0925	0.1432	6.5633	5.7833	7.2361	6.0725	6.8915	4.3761
2022008749	35.33	0.0501	0.1155	8.8867	7.9067	8.7476	8.3020	7.3310	6.7171
2022011641	22.37	0.0431	0.0611	8.6300	8.3567	10.4046	8.7745	9.0615	7.2132
2022011769	32.4	0.0578	0.0376	8.6733	8.4700	9.5624	8.8935	5.7737	7.3382
2022012068	31.82	0.0787	0.0909	2.4600	4.7333	1.3788	4.9700	2.5830	5.2185
2022012664	34.11	0.0685	0.0951	5.3700	8.6700	5.9204	9.1035	5.6385	9.5587
2022013221	28.6	0.0711	0.1324	7.9400	8.5633	8.7539	8.9915	8.3370	9.4411
2022015878	32.82	0.0815	0.1204	3.1433	4.8633	-2.5345	2.1065	3.3005	5.3618
2022002594	46.29	0.0654	0.0889	9.4500	8.2267	10.4186	8.6380	9.9225	9.0699
2022003618	27.57	0.0442	0.0794	10.0900	8.3767	11.1242	8.7955	10.5945	9.2353
2022006807	22.37	0.0328	0.0848	8.0433	5.7533	10.2011	6.0410	8.4455	6.3431
2022008054	16.82	0.0556	0.1088	8.3600	2.9433	9.2169	1.0905	8.7780	4.2450
2022008447	35.35	0.0222	0.0522	9.6733	7.6733	7.0901	8.0570	7.7525	6.4599
2022011541	36.91	0.0457	0.0583	8.5767	6.9000	7.8558	7.2450	9.0055	7.6073
2022011981	23.72	0.0246	0.0692	10.9000	8.7800	12.0173	9.2190	12.4450	9.6800
2022011983	24.2	0.0189	0.0527	9.4267	5.9867	10.3929	6.2860	9.8980	6.6003
2022013628	29.52	0.0745	0.1124	8.3233	5.6867	9.1765	5.9710	8.7395	6.2696
2022016533	46.96	0.0533	0.1083	9.2900	6.2900	10.2422	6.6045	9.7545	6.9347
2022015396	30.54	0.0779	0.0733	7.9467	4.9533	8.7612	5.2010	8.3440	5.4611

Table S3 (Related to Figure 1) Clinical data of 20 patients with healthy subjects

Case#	Sex	Age	Serum (μmol/L)
2024024380	Female	42	64.3
2024023866	Female	52	46.8
2024024425	Female	34	45.5
2024024038	Female	34	47.2
2024023831	Female	36	61.1
2024024350	Female	30	48.2
2024024732	Female	49	55.4
2024024748	Female	55	80.2
2024024845	Female	26	54
2024024961	Female	69	40.9
2024025071	Male	23	47.4
2024025044	Male	29	39.8
2024025050	Male	59	56.4
2024025310	Male	37	37.4
2024025254	Male	57	48.8
2024025282	Male	58	44.2
2024025257	Female	45	44.2
2024025350	Female	54	39.5
2024025735	Female	49	53.4
2024025709	Female	50	52.2

Table S4 (related to Figure 1) Plasma amino acid in Healthy subjects (n=20)

	#1	#2	#3	#4	#5	#6	#7	#8	#9	#10	#11	#12	#13	#14	#15
Ala (μmol/L)	703.80	400.30	597.10	305.10	582.90	383.10	533.30	420.00	299.50	435.80	275.00	317.80	578.70	348.30	262.50
Glu (μmol/L)	268.80	226.50	250.70	228.90	240.00	256.40	206.10	217.80	159.80	237.70	190.00	198.70	262.70	184.10	185.30
Val (μmol/L)	303.70	249.50	220.10	215.80	207.70	321.10	202.60	305.80	236.30	244.80	261.10	221.40	259.00	242.20	259.70
Gly (μmol/L)	275.00	235.50	186.10	293.90	171.90	207.70	294.70	357.70	171.10	191.90	358.70	290.00	271.00	174.00	394.40
Thr (μmol/L)	161.00	111.70	123.70	84.10	165.10	144.50	129.40	159.40	78.40	75.80	203.20	121.80	163.20	113.80	87.10
Ser (μmol/L)	122.10	101.30	122.60	124.40	90.90	142.20	123.20	179.60	113.70	129.30	183.60	150.40	130.70	127.00	133.20
Pro (μmol/L)	243.00	111.70	153.80	123.50	186.80	189.10	166.90	177.00	121.50	121.80	115.40	205.70	216.10	194.70	124.50
Orn (μmol/L)	125.70	118.90	86.70	67.00	71.60	78.20	82.40	114.70	52.50	69.10	63.00	68.50	110.20	56.30	86.70
Arg (μmol/L)	81.40	68.70	94.90	56.10	86.60	95.90	70.00	90.80	54.60	76.20	71.30	73.60	95.70	81.30	62.90
Lys (μmol/L)	260.30	191.90	187.90	130.70	207.60	170.60	200.30	247.50	168.70	223.90	164.10	196.70	265.10	175.60	148.50
Leu (μmol/L)	155.30	125.50	146.90	110.70	121.50	162.70	129.80	169.10	141.70	143.00	131.70	117.40	153.80	102.90	138.70
His (μmol/L)	78.10	80.90	92.20	66.80	90.30	78.20	88.60	77.70	83.40	79.20	77.20	75.10	88.10	72.20	75.10
Phe (μmol/L)	73.60	54.50	71.00	65.00	62.10	44.00	61.30	79.80	65.90	64.80	52.60	51.20	83.60	51.90	58.70
Tyr (μmol/L)	112.40	63.40	73.10	55.10	96.00	72.00	74.80	77.90	53.80	67.40	56.60	63.60	59.70	44.00	66.90
Cys (μmol/L)	25.70	29.20	18.40	19.60	18.20	31.40	28.50	49.10	17.70	25.80	26.10	31.00	38.40	28.40	29.20
Met (μmol/L)	30.10	23.50	27.20	25.70	30.00	25.00	28.50	46.30	24.50	24.50	20.30	27.80	37.30	20.60	17.20
Ile (μmol/L)	76.40	49.60	75.30	53.30	51.30	84.50	57.60	107.30	69.90	68.80	69.40	63.10	81.80	55.50	70.80
Tau (μmol/L)	68.20	60.40	38.20	48.00	49.20	35.30	37.30	63.90	56.90	90.90	51.20	46.00	38.50	41.30	35.50
Trp (μmol/L)	64.30	46.80	45.50	47.20	61.10	48.20	55.40	80.20	54.00	40.90	47.40	39.80	56.40	37.40	48.80
Asp (μmol/L)	14.90	15.80	15.50	12.00	15.70	14.40	14.50	17.00	11.70	11.50	14.20	13.00	17.80	11.90	13.30

#16	#17	#18	#19	#20
362.90	449.50	336.40	538.70	547.20
185.30	201.80	177.40	186.90	217.40
233.20	277.90	181.60	249.70	265.20
320.60	201.40	458.90	385.20	173.40
140.20	95.40	188.90	188.40	104.70
118.70	96.50	195.40	168.10	80.40
141.10	140.50	193.10	184.90	186.10
67.70	57.30	93.30	73.70	93.20
59.40	60.70	70.00	105.00	72.00
213.60	197.20	169.00	206.80	197.60
112.80	120.50	103.60	121.90	127.50
73.50	79.50	84.40	83.40	86.50
56.00	46.30	55.40	61.50	52.20
55.50	44.30	46.00	77.70	58.80
35.20	21.20	36.10	29.30	31.90
19.80	18.00	22.60	32.80	22.20
58.10	61.70	48.10	63.70	75.70
54.30	33.50	38.10	60.20	60.40
44.20	44.20	39.50	53.40	52.20
10.30	12.10	12.80	15.20	10.50

Table S5 (related to Figure 1) Plasma amino acid in patients with conlon cancer (n=20)

	#1	#2	#3	#4	#5	#6	#7	#8	#9	#10	#11	#12	#13
Ala (μmol/L)	219.42	451.94	398.08	336.36	406.92	230.77	252.98	414.91	378.18	330.40	436.89	369.30	346.28
Glu (μmol/L)	107.03	99.24	94.47	158.85	162.53	181.50	71.38	138.70	121.44	117.27	115.37	113.14	94.23
Val (μmol/L)	471.76	309.30	215.38	438.28	294.77	375.11	231.50	242.86	510.50	427.09	443.24	313.45	398.63
Gly (μmol/L)	432.76	683.58	383.22	270.59	284.28	312.58	194.52	326.70	338.91	414.23	243.46	241.94	271.33
Thr (μmol/L)	108.87	202.73	209.25	181.44	127.38	92.30	72.52	143.81	136.95	112.98	127.12	130.81	102.85
Ser (μmol/L)	169.83	309.89	215.74	203.51	147.74	197.75	101.00	193.60	193.05	206.44	150.43	163.93	138.57
Pro (μmol/L)	216.58	223.12	147.34	263.07	585.21	174.66	103.04	188.05	256.56	137.65	207.80	192.86	182.04
Orn (μmol/L)	67.84	47.11	37.22	63.58	47.59	71.95	15.75	95.64	111.85	36.21	47.51	186.67	39.12
Arg (μmol/L)	72.76	147.48	112.52	109.85	86.74	96.40	51.51	143.84	129.79	88.84	140.05	74.66	82.63
Lys (μmol/L)	186.17	279.01	286.28	348.52	241.82	256.50	146.81	278.47	257.33	227.00	290.77	240.49	244.34
Leu (μmol/L)	213.55	263.21	139.02	269.70	250.85	236.34	59.26	145.39	267.57	134.06	211.15	231.15	157.69
His (μmol/L)	73.40	93.61	73.80	66.43	102.48	82.09	52.99	100.41	102.25	66.36	100.27	89.08	89.77
Phe (μmol/L)	44.66	60.98	47.14	53.45	56.27	44.62	32.04	51.90	71.33	44.46	67.58	64.22	62.64
Tyr (μmol/L)	40.68	66.16	60.23	68.92	52.79	51.24	30.81	62.74	57.58	12.20	48.18	59.63	51.66
Cys (μmol/L)	57.98	29.97	14.99	53.40	39.34	28.03	23.99	45.69	46.75	17.06	29.47	41.01	25.18
Met (μmol/L)	21.99	41.95	20.24	37.01	26.00	14.89	37.22	126.23	127.74	75.10	24.25	80.06	90.28
Ile (μmol/L)	104.83	112.96	77.55	174.25	104.26	137.17	50.23	87.01	178.27	83.18	129.82	112.82	89.57
Tau (μmol/L)	10.14	71.10	35.55	17.22	59.53	19.59	26.29	51.43	59.50	45.60	43.75	54.85	30.94
Trp (μmol/L)	17.03	26.96	32.40	35.35	15.33	27.57	16.82	24.20	31.82	28.60	36.91	29.52	23.72
Asp (μmol/L)	3.33	1.92	2.80	0.48	0.94	2.49	1.41	2.37	4.03	3.77	3.54	3.00	2.24

#14	#15	#16	#17	#18	#19	#20
380.09	611.37	200.51	363.43	394.57	300.06	539.97
75.17	118.37	37.89	172.47	167.72	177.37	152.50
184.85	270.62	151.50	413.03	200.28	602.23	321.51
373.31	315.08	163.56	247.24	567.32	322.45	275.88
150.21	104.39	61.97	153.64	130.94	141.88	163.19
188.56	180.98	88.24	215.19	258.70	194.60	173.80
412.21	188.45	96.45	214.53	222.29	230.14	238.21
44.67	42.45	2.43	80.23	70.39	71.66	167.42
114.46	106.49	42.02	104.02	139.80	104.20	89.71
223.58	284.78	78.64	271.82	282.18	284.04	336.25
116.30	146.76	94.18	211.16	118.52	498.38	171.51
81.21	98.29	63.00	85.43	72.73	94.05	79.62
51.63	47.57	40.56	44.96	47.02	55.38	48.83
55.15	54.98	39.05	51.13	56.04	44.52	61.68
38.91	12.88	5.40	38.05	33.23	48.98	10.70
23.21	87.31	45.81	94.37	17.92	26.04	22.44
86.41	79.17	43.30	150.06	117.71	220.46	109.18
54.31	29.87	41.70	45.02	42.59	33.36	64.65
30.54	46.29	21.95	22.37	42.80	34.11	32.82
1.69	4.31	2.04	4.03	1.51	1.76	1.04

Table S6 (related to Figure 1) MiRNA profile associated to tryptophan

ID	Trp- (log2)	Trp- (log2)	Trp+ (log2)	Trp+ (log2)
mmu-miR-15b-5p	1.96	1.14	5.06	5.19
mmu-miR-23b-3p	7.51	6.96	8.73	9.11
mmu-miR-27b-3p	1.93	1.93	5.22	5.56
mmu-miR-30a-5p	1.91	1.62	2.93	3.95
mmu-miR-125a-5p	4.54	3.62	7.8	8.08
mmu-miR-125b-5p	6.35	6.37	8.68	9.28
mmu-miR-145a-5p	2.13	1.33	6.12	6.2
mmu-miR-149-5p	1.29	0.81	3.21	3
mmu-miR-151-3p	1.25	1.32	2.42	2.83
mmu-miR-181a-5p	1.14	1	2.53	3.14
mmu-miR-185-5p	1.3	1.85	2.93	3.38
mmu-miR-193a-3p	1.52	2.08	3.57	3.56
mmu-miR-199a-5p	1.06	0.95	2.85	4.16
mmu-miR-106a-5p	0.91	2.05	3.8	5.31
mmu-miR-19b-3p	1.57	1.76	4.03	5.86
mmu-miR-30c-5p	2.36	2.1	4.65	7.65
mmu-miR-16-5p	6.3	1.12	7.92	2.53
mmu-miR-18a-5p	0.98	1.5	2.29	5.57
mmu-miR-20a-5p	2.08	2.43	5.98	5.99
mmu-miR-22-3p	2.39	5.73	5.38	8.57
mmu-miR-23a-3p	6.03	3.74	8.07	6.03
mmu-miR-26a-5p	6.49	3.18	7.61	5.72
mmu-miR-29a-3p	3.49	8.47	5.38	10.01
mmu-miR-27a-3p	3.4	0.82	5.51	3.31
mmu-miR-31-5p	8.48	2.36	9.83	7.37
mmu-miR-31-3p	0.98	1.95	2.85	4.75
mmu-miR-92a-3p	3.66	5.19	7.19	7.07
mmu-miR-93-5p	3.61	4.58	4.96	6.93
mmu-miR-103-3p	5.4	1.43	6.91	6.98
mmu-miR-345-3p	1.39	0.9	2.62	3.7
mmu-miR-107-3p	4.94	1.67	6.69	5.62
mmu-miR-17-5p	3.07	2.34	6.85	6.94
mmu-miR-25-3p	1	1.43	2.22	3.01
mmu-miR-221-3p	1.37	1.46	5.52	4.33
mmu-miR-222-3p	4.64	3.52	6.81	5.56
mmu-miR-125b-1-3p	1.06	1.59	3.76	3.46
mmu-miR-361-5p	1.57	1.82	3.14	3.65
mmu-miR-378a-3p	4.05	3.14	5.1	5.62
mmu-miR-410-5p	0.78	1.8	1.93	4.35
mmu-miR-486-5p	1.03	4.63	2.11	8.39
